# Architectural confinement and seasonal forcing shape cross-domain pathogen-associated assemblages in complex built environments

**DOI:** 10.1128/spectrum.01915-26

**Published:** 2026-08-31

**Authors:** Shijiao Qi, Shurui Zhang, Yixin Hu, Mingjie Sun, Zichun Xing, Shanshan Bao, Yuyang Song, Li Sun, Xinzhao Tong

**Affiliations:** 1 Department of Biosciences and Bioinformatics, School of Science, Xi'an Jiaotong-Liverpool University 668947, Suzhou, People's Republic of China; Sichuan University, Chengdu, China

**Keywords:** built environment microbiome, pathogen-associated taxa, cross-domain associations, spatiotemporal dynamics, microbial ecology

## Abstract

Airborne microbial pathogens in built environments (BEs) may pose health threats, yet the ecological mechanisms governing their assembly and persistence across interconnected architectural spaces are less studied. Here, we conducted a year-long, multi-spatial investigation across a university building complex, sampling the exhaust outlets of three indoor environments and adjacent outdoor inlets. By integrating the 16S rRNA gene and ITS sequencing from 437 paired bacterial–fungal samples, we characterized the spatiotemporal dynamics of airborne opportunistic pathogen-containing genera. Our study showed that spatial filtering emerged as the dominant determinant of pathogen community structure, with confined elevator environments serving as reservoirs of potential pathogens, with limited but continuous microbial influx from surrounding spaces. Some potential pathogens exhibited distinct seasonal dynamics, as exemplified by pathogen-associated fungal genera such as *Fusarium*, which peaked in autumn and winter, possibly driven by enhanced aerosol persistence and dispersal under cooler, drier conditions. In contrast, the bacterial counterparts exhibited greater temporal resilience, with key taxa such as *Listeria* actively transcribed during winter, predisposing them to increased relative abundance in spring. Cross-domain ecological networks further revealed dynamic associations among potential pathogens, centered on the skeletal structure mediated by the keystone fungal genus *Aspergillus*, suggesting that coordinated microbial associations may reinforce their persistence across seasons. Together, our findings suggest that the dynamics of pathogen-containing genera within BEs arise from the coupled effects of spatial filtering, climatic modulation, and microbial associations. These results provide a foundation for transitioning from static environmental control toward predictive pathogen management in BEs.

**IMPORTANCE:**

Built environments (BEs) are the primary settings of human microbial exposure, yet the ecological principles governing the persistence of airborne pathogens across interconnected indoor spaces remain poorly resolved. By integrating bacterial and fungal community dynamics across spatial environments over four seasons, this year-long study demonstrates that the ecology of airborne pathogen-associated taxa is not static but is instead mediated by a complex interplay of spatial, climatic, and biological forces. Our findings identify enclosed, high-transit elevator spaces as critical hotspots for the accumulation of potential pathogens and highlight the role of seasonal ecological reorganization in driving airborne health risks. More broadly, this work establishes a system-level ecological framework for understanding the dynamics of airborne pathogen-associated taxa in BEs and provides a conceptual basis for developing more adaptive strategies for indoor microbial risk management.

## INTRODUCTION

With rapid urbanization in the 21st century, increasingly complex built environments (BEs) have become essential to support dense populations and expanding infrastructure systems. As a consequence, modern human activities are overwhelmingly confined to indoor spaces, including residential, commercial, and transportation settings (1). Despite routine cleaning and disinfection, these environments are far from sterile. Instead, they harbor diverse and dynamic airborne microbial communities composed of bacteria, fungi, and other microorganisms. Among these, exposure to a subset of opportunistic pathogens may pose substantial health risks through inhalation and deposition in the respiratory tract. Common airborne bacterial genera, such as *Legionella*, *Mycobacterium*, *Staphylococcus*, and *Pseudomonas*, and fungal taxa, including *Aspergillus*, *Cladosporium*, *Penicillium*, and *Alternaria*, are frequently detected in BEs and have been associated with respiratory infections, allergic diseases, and asthma exacerbation (2).

The presence of these microorganisms is not random but governed by ecological processes that regulate their composition and persistence. Airborne microbial communities in BEs are shaped by the combined effects of environmental selection and dispersal dynamics (2). Environmental and building factors, including temperature, relative humidity, ventilation rate, light availability, and building materials, act as selective filters that determine microbial survival, growth, and activity (3). At the same time, dispersal processes mediated by airflow, human movement, and architectural connectivity influence microbial transmission across spaces (2). Together, these processes generate spatially heterogeneous microbial communities within buildings.

Such heterogeneity is particularly evident across distinct architectural environments. Underground spaces are typically characterized by elevated humidity, more stable temperatures, limited light, and restricted ventilation, which may serve as reservoirs for humidity-associated pathogens, including toxigenic fungi, that can be redistributed via ventilation-mediated transport (4). In contrast, aboveground indoor environments are more strongly influenced by outdoor air exchange and diurnal environmental fluctuations. Confined microenvironments, such as elevators, represent highly connected nodes with heavy human traffic, frequent surface contact, and limited air circulation, creating unique ecological conditions for microbial accumulation and redistribution. These differences not only shape microbial diversity but also influence the persistence and transmission potential of pathogens (5). Therefore, enclosed and high-traffic spaces may facilitate microbial homogenization and act as hubs for pathogen accumulation and dispersal, thereby increasing the likelihood of aerosol transmission through shared airflows and ventilation systems (5).

Beyond spatial heterogeneity, temporal variability further modulates microbial dynamics. Seasonal climatic fluctuations alter environmental conditions governing microbial growth and dispersal (6), an effect especially pronounced in subtropical monsoon regions, where temperature and humidity vary substantially across seasons. The Yangtze River Delta, one of the most densely populated and rapidly urbanized regions in China, exemplifies this climatic regime. A defining feature of this region is the annual plum rain season, characterized by persistent precipitation, elevated humidity, and warm temperatures during June and July (7). These conditions sustain high indoor moisture levels and create favorable environments for microbial proliferation, particularly for fungi and moisture-associated bacterial taxa (2). Although climatic influences on indoor microbiomes have been widely recognized, the interactive effects of spatial heterogeneity and seasonal climatic variability across vertically connected public BEs remain insufficiently characterized.

In this study, we conducted a year-long investigation (September 2023 to August 2024) on a university campus in the Yangtze River Delta, serving as a model system for complex public buildings with interconnected, functionally distinct compartments. We continuously monitored temperature and relative humidity across four spatial settings: aboveground indoor spaces, underground facilities, elevators, and outdoor environments. Concurrently, bacterial and fungal communities were characterized using 16S rRNA gene (16S rDNA), 16S rRNA, and ITS sequencing of swab samples collected from the outlets and inlets of the ventilation systems. A total of 437 paired bacterial–fungal samples were obtained, enabling a comprehensive assessment of microbial diversity and composition across spatial and temporal gradients.

This study aims to (i) characterize the spatiotemporal dynamics of bacterial and fungal communities across distinct spatial environments, (ii) identify key environmental drivers shaping microbial diversity, composition, and metabolic activity, (iii) evaluate the distribution and dispersal potential of pathogen-associated taxa across interconnected indoor spaces, and (iv) explore the associations between bacterial and fungal taxa, with particular emphasis on co-occurrence patterns among microbes with pathogenic potential. By integrating microbial ecology with architectural and climatic factors, understanding these dynamics is essential for designing healthier BEs and mitigating the risk of airborne pathogen transmission.

## MATERIALS AND METHODS

### Study design

This study was conducted in the Science Buildings of Xi’an Jiaotong-Liverpool University (Suzhou, Jiangsu Province, China), which were selected for their extensive subterranean infrastructure encompassing parking lots, corridors, restrooms, offices, and a canteen. These underground environments were characterized by poor natural lighting, limited ventilation, and high human occupancy, providing an ideal setting to investigate microbial dispersal dynamics across interconnected BEs. We hypothesized that outdoor-sourced microbes infiltrate indoor spaces (both underground and aboveground) through ventilation systems and open architectural features (e.g., windows), and indoor microbes disperse vertically through air currents and human movements, facilitated by elevators and shared corridors. To test these hypotheses, we stratified 44 ventilation system sites into four categories based on their spatial distribution: (i) underground ventilation outlets (*n* = 28 sites): parking lots, restrooms, corridors, a canteen, and offices; (ii) aboveground ventilation outlets (*n* = 9 sites): offices, laboratories, classrooms, and restrooms on floors 2–5; (iii) elevator ventilation outlet (*n* = 4 sites): four elevators connecting underground and aboveground floors; (iv) outdoor ventilation inlets (*n* = 3 sites): air intake vents for outdoor microbial inputs.

### Sample collection and sequencing

Sampling was conducted every 2 weeks from September 2023 to August 2024, excluding an 8-week university closure during early January and late February 2024 (Fig. S1a), resulting in a total of 21 sampling events. Each month, two sampling rounds were performed: the first targeted 22 distinct sites (represented by a suffix “A” in sample names in Fig. S1b), and the second targeted the other 22 sites (represented by a suffix “B” in sample names in Fig. S1b). During the sampling period, the summer months (June to August) exhibited peak warmth and moisture, with a marked spike in humidity during the plum rain season (June to July), whereas winter (December to February) remained cold and dry (Fig. S1c and d). Additionally, we collected 36 samples for 16S rRNA sequencing from underground areas in two contrasting seasons (i.e., winter and plum rain summer). Although we initially designed the collection of fungal rRNA samples, these samples failed to yield sufficient high-quality sequencing data. During each sampling event, environmental parameters, including temperature and relative humidity, were measured at each site using a portable environmental sensor, and the architectural metadata, including site location, building function, and floor level, were carefully documented.

Biological samples were collected from the outlet surfaces of ventilation systems in three indoor spaces (i.e., underground, aboveground, and elevators) and outdoor inlet vents using Copan eSwabs pre-moistened with 1 mL liquid Amies preservation medium to maximize biomass recovery. During winter and plum rain summer seasons, two parallel samples were collected for 16S rDNA and rRNA sequencing, respectively. To preserve RNA integrity, the swabs collected for rRNA sequencing were immediately transferred to tubes containing DNA/RNA Shield solution (Zymo Research, United States). All samples were stored at −80°C within 2 h of collection and processed accordingly.

Genomic DNA (gDNA) was extracted from the preservation solution using the QIAGEN DNeasy PowerSoil Pro Kit with optimized protocols to enhance extraction yield and purity. Modifications included (i) addition of 500 μL culture medium to bead-containing centrifuge tubes to improve cell lysis, (ii) rigorous supernatant transfer to maximize DNA recovery, and (iii) implementation of a two-step C5 solution wash (300 μL × 2) to reduce co-purified impurities. All lysates were filtered through an MB spin column according to the manufacturer’s instructions. DNA concentrations were quantified using a Qubit fluorometer (Thermo Fisher Scientific, USA) with high-sensitivity dsDNA assays. Following the manufacturer’s protocols, samples were incubated with a fluorescent dye to form DNA-dye complexes, and triplicate measurements were taken to ensure quantification accuracy. Only extracts meeting the minimum concentration threshold (≥0.5 ng/µL) were processed for downstream sequencing.

Total RNA was extracted from parallel samples using the QIAGEN RNeasy PowerSoil Total RNA Kit according to the manufacturer’s guidelines. The residual gDNA in RNA extracts was digested using the TURBO DNA-free Kit (Thermo Fisher Scientific, United States). The purified total RNA was then reverse transcribed into complementary DNA (cDNA) using SuperScript III reverse transcriptase (Invitrogen, Carlsbad, CA), as described previously (8).

The preparation of gDNA and cDNA amplicon libraries was performed using primers 515F (5′-GTGCCAGCMGCCGCGGTAA-3′) and 806R (5′-GGACTACHVGGGTWTCTAAT-3′), targeting the bacterial 16S rRNA V4 hypervariable region. The fungal ITS1 region was amplified using the primer pairs ITS1-F (5′-CTTGGTCATTTAGAGGAAGTAA-3′) and ITS1-R (5′-GCTGCGTTCTTCATCGATGC-3′). Libraries were sequenced on an Illumina MiSeq platform (2 × 250 bp paired-end) by Novogene (Beijing, China), generating over 50,000 paired-end reads for most of the samples. Fourteen samples without sufficient amplicon libraries failed during sequencing, leaving raw sequences for 437 paired bacterial–fungal sample groups and 36 bacterial rRNA samples. To mitigate contamination, four unused swabs were processed in parallel with the samples and included as negative controls. All samples were sequenced in a single batch to minimize technical variability.

### QIIME2-based amplicon sequencing data processing workflow

Raw sequences were processed using the QIIME2 platform (v2024.2) (9). After importing the sequence files, DADA2 (10) was used to denoise the data and generate a feature table. The representative sequences (i.e., features) were clustered into amplicon sequence variants (ASVs), yielding 142,540 high-quality ASVs from the bacterial rDNA and rRNA samples and 45,642 from the fungal rDNA samples. ASVs in the negative controls were considered contaminants and identified using the QIIME2 function “decontam.” Following the removal of contaminant ASVs, the Greengenes2 database (v2024.09) (11) was used to classify clean bacterial ASVs from the phylum to genus levels. Taxonomic classification of fungal ASVs was performed using the feature-classifier classify-sklearn command against the UNITE database (v9.0) (12) tailored for ITS-based fungal identification. This classification process assigned taxonomic labels from phylum to genus, based on sequence similarity to reference sequences.

### Diversity analyses

The bacterial and fungal ASV count tables, taxonomy annotation table, and sample metadata were integrated into a single object using the R package “phyloseq” (v1.50.0) (13). Before performing the diversity analysis, bacterial samples were rarefied to an even depth of 20,573 reads (Fig. S2a), and fungal samples were rarefied to 40,000 (Fig. S2b). After rarefaction, 20 bacterial and two fungal samples with insufficient reads were discarded. Subsequently, the rarefied bacterial and fungal ASV count tables were used in the diversity analyses. Shannon diversity and richness were used as metrics for alpha-diversity inference. The significance of season and spatial types on alpha diversity of bacterial and fungal communities was studied using the Kruskal-Wallis test. Pairwise comparisons were conducted using the post hoc Wilcoxon test with the Benjamini-Hochberg correction to control for false discovery rate (FDR).

To further characterize temporal dynamics in alpha diversity, generalized additive models (GAMs) were fitted for log-transformed bacterial and fungal richness using the “mgcv” package (v1.9-4) in R. Using a hierarchical smooth framework, temporal variation was partitioned into long-term trends and seasonal cycles. Long-term trends (non-cyclical structural fluctuations) were modeled using a thin-plate regression spline in cumulative sampling time (i.e., days), with group-specific smooths to capture deviations. In contrast, seasonal variation (recurring annual cycles) was modeled using a cyclic cubic regression spline on day of year, likewise with group-specific smooths, while enforcing continuity between the start and end of the year. Temperature and relative humidity were included as linear covariates after *z*-score standardization. Models were fitted using restricted maximum likelihood and evaluated using adjusted *R*^2^ and explained deviance. Finally, the relative contributions of long-term trends, seasonal cycles, group deviations, and climatic factors to the variance were quantified by sequential component removal.

For beta-diversity analysis, patterns in community composition across treatment groups were evaluated using abundance-based Bray–Curtis dissimilarity for both bacterial and fungal ASVs and subsequently visualized via principal coordinates analysis (PCoA). The significance of building and temporal factors on the beta-diversity was assessed using permutational multivariate analysis of variance (PERMANOVA) (14). In addition, permutational analysis of multivariate dispersions (PERMDISP) (15) was applied to study the homogeneity of dispersion among samples from different spatial groups and seasons. PERMANOVA and PERMDISP analyses were performed using the R package “vegan” (v2.7.3).

### Pathogen inference and community-level analysis

The list of pathogen taxa was downloaded from the Global Catalog of Pathogens (gcPathogen) database (16) on 3 April 2024. Following the CDC/NIH Biosafety in Microbiological and Biomedical Laboratories guidelines, only bacterial or fungal species classified as biosafety level (BSL) 2 or above were considered potential human pathogens (17). This threshold focuses the analysis on microorganisms posing moderate-to-high hazards to personnel and the environment. Since 16S rRNA and ITS gene sequences provide reliable taxonomic resolution at the genus level but are less so at the species level (18), we extracted genus-level information from the bacterial and fungal pathogen lists and matched it with our taxonomy tables, defining these as pathogen-containing genera. To address multi-species genera with mixed pathogenicity, a conservative approach was applied: a genus was classified as pathogen containing if it harbored at least one validated human-pathogenic species at BSL-2 or higher. While this threshold may overestimate pathogenic potential, it ensures that no recognized risk is overlooked, given that pathogenicity is often not uniform across all congeneric species. To ensure methodological consistency, alpha- and beta-diversity analyses for the pathogen-associated communities were determined via the Shannon index and Bray–Curtis dissimilarity, respectively, following the protocols established for the total communities.

### Influence of environmental parameters on the microbial occurrence pattern

MaAsLin3 (v0.99.16) (19) was used to assess how environmental parameters (i.e., temperature and relative humidity) affect the occurrence patterns (e.g., relative abundance and occurrence frequency) of bacteria and fungi, particularly pathogen-associated genera. Spearman’s rank correlation revealed a significant correlation between temperature and relative humidity, with a coefficient (ρ) value of 0.627. Thus, temperature and relative humidity, as independent factors, and their interaction were also included as predictors, and the relative abundance of bacterial and fungal genera was used as the response variable in a linear mixed-effects regression model. Additionally, a logistic regression model was applied, with the prevalence of bacterial and fungal genera serving as the response variable. The FDR correction was used to adjust for multiple comparisons; by default, an FDR-corrected *P*-value (i.e., *q*-value) <0.1 is considered significant.

### Transcriptional activity of bacterial taxa

All the rDNA and rRNA bacterial samples were initially rarefied to a depth of 20,573 reads per sample, as indicated above. For a given rDNA sample, the relative transcriptional activity of an ASV in the rDNA sample can be estimated by calculating the count number of this ASV in the corresponding rRNA samples, divided by its count number in the rDNA sample. Based on a previous study (8), the metabolically active bacterial taxa are defined as those with an rRNA/rDNA ratio >1, and those with a ratio between 0 and 1 are considered metabolically inactive. The rRNA/rDNA ratio of each ASV in winter and plum rain summer seasons was compared to infer bacterial metabolic activity under contrasting conditions.

### Microbial dispersal prediction

The Sloan neutral community model (NCM) (v2015.10) (20) was applied to simulate the diffusion of individual bacteria and fungi from outdoor to indoor spaces and their dispersal within indoor environments. In this model, the outdoor samples were treated as sources, and the indoor samples were treated as sinks. Within indoor environments, the three space types (i.e., underground, aboveground, and elevators) were used as sources and sinks, respectively, to understand bidirectional dispersal between indoor spaces. The rarefied bacterial and fungal ASV count tables were used in the NCM, respectively, and we assumed that ASVs with a higher relative abundance in the source are more likely to spread to the sink by chance, while the rare ones are more prone to be lost during dispersal due to ecological drift (21). The goodness of fit of the NCM was assessed using the coefficient of determination (*R*^2^), with a range from ≤0 (no fit) to 1 (perfect fit). The estimated migration rate was represented by the parameter *m*. All ASVs in each prediction case were sorted into the three partitions based on whether and how they deviated from the 95% CI around the neutral model prediction: above-neutral (occurred more frequently and/or at greater abundance than predicted by the neutral model), below-neutral (occurred less frequently and/or at lower abundance than predicted), or neutral (within prediction). Only ASVs that fall within the above-neutral and neutral partitions were considered to be capable of dispersal.

### Cross-domain microbial network construction

Since bacteria and fungi are both important members of indoor microbiota, we constructed cross-domain microbial networks to understand their associations, using the SPIEC-EASI algorithm (22). To minimize the noise from the rare taxa, only the top 200 most abundant bacterial and fungal ASVs were retained for network analysis. Crucially, while these network edges capture statistical associations, they do not necessarily imply direct ecological interactions. To ensure robustness, only associations with an absolute Spearman’s correlation coefficient ≥0.6 were retained for visualization. Network construction and visualization were conducted in R using the “ggClusterNet” package (v2.0).

### Bacterial–fungal coupling analysis

Community concordance between bacteria and fungi was evaluated across 437 paired samples by using Procrustes analysis (the “protest” function) on their respective Bray–Curtis dissimilarity matrices. The strength and significance of the cross-domain association were determined using the Procrustes correlation coefficient (*r*) and permutation-based *P*-values. Complementary to the Procrustes analysis, Mantel tests were performed to assess correlations between bacterial and fungal dissimilarity matrices using Spearman’s rank correlation. Procrustes residuals, representing sample-wise dissimilarity between bacterial and fungal ordinations, were extracted and used as a quantitative measure of coupling strength. Differences in residuals across environmental groups and seasons were assessed using analysis of variance (ANOVA). Procrustes, Mantel, and ANOVA tests were all performed using the R package “vegan.”

## RESULTS

### Spatiotemporal dynamics of bacterial and fungal communities

**Fig 1:**
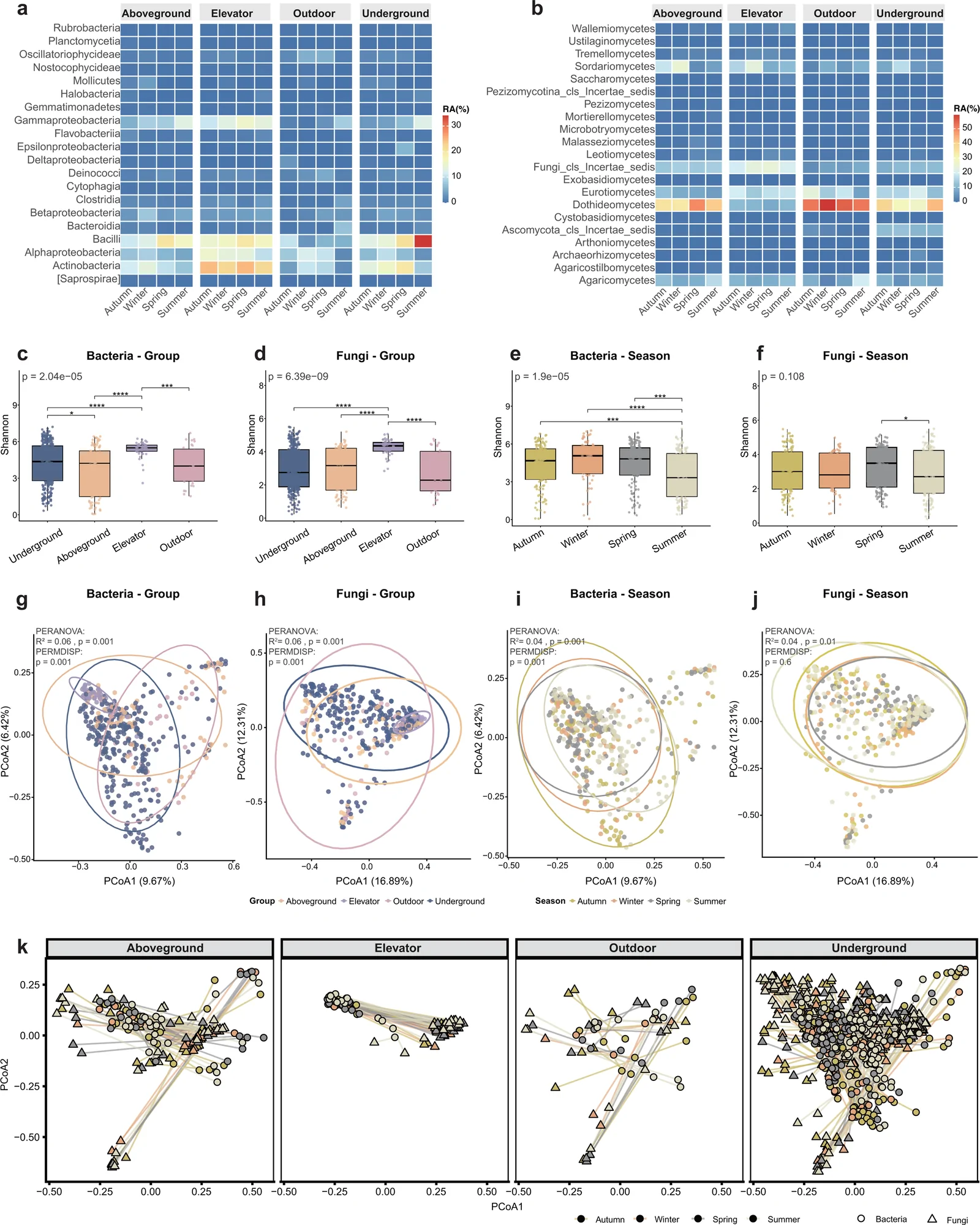
Spatial and seasonal structuring of airborne microbial communities. (**a and b**) Relative abundances of the top 20 bacterial (**a**) and fungal (**b**) classes across all samples. (**c and d**) Shannon diversity of bacterial (**c**) and fungal (**d**) communities across four spatial environments. (**e and f**) Shannon diversity of bacterial (**e**) and fungal (**f**) communities across four seasons. (**g and h**) Principal coordinates analysis (PCoA) of bacterial (**g**) and fungal (**h**) communities across spatial environments, using Bray–Curtis dissimilarity for both domains. Ellipses represent 95% confidence intervals for group centroids. (**i and j**) PCoA of bacterial (**i**) and fungal (**j**) communities across seasons using the same dissimilarity metrics as indicated above. (**k**) Procrustes-based coupling between bacterial and fungal community structures along spatial gradients, indicating coordinated shifts across environments.

At the community level, bacterial assemblages were consistently dominated by four major classes throughout all seasons: Bacilli, Actinobacteria, Alphaproteobacteria, and Gammaproteobacteria (Fig. 1a). However, their temporal dynamics showed a spatially dependent pattern. In elevators, these taxa maintained relatively stable abundances across seasons, with Gammaproteobacteria and Actinobacteria particularly enriched. In contrast, aboveground and underground environments exhibited pronounced seasonal variation: Bacilli gradually accumulated from autumn to summer in underground spaces, whereas in aboveground settings, they peaked in spring before declining. Compared to the bacterial community, the fungal counterpart displayed markedly different, less diverse class-level patterns. Dothideomycetes dominated outdoor, aboveground, and underground environments, but were disproportionately depleted in elevators (Fig. 1b). Furthermore, Eurotiomycetes were less abundant in aboveground settings than the other spatial types, and Sordariomycetes showed consistent winter peaks across all spatial types.

The overall Shannon diversity of bacterial and fungal communities was positively correlated (Spearman’s ρ = 0.681, $P\;=\;5.65\times 10^{-34}$), suggesting partially shared ecological drivers across time and space (Fig. S3). When spatial setting was considered, significant differences in alpha diversity were detected for both domains (Kruskal-Wallis test, $P_{\text{bacteria}}\;=\;2.04\times 10^{-5}$, $P_{\text{fungi}}\;=\;6.39\times 10^{-9}$), following a parallel gradient in which elevator environments consistently harbored the highest diversity, while outdoor settings exhibited the lowest diversity (Fig. 1c and d). Conversely, temporal dynamics diverged between domains. Bacterial diversity differed significantly across seasons ($P=1.90\times 10^{-5}$), peaking in winter and declining in summer, whereas fungal diversity showed no statistically significant seasonal changes (P=0.108), though there was a tendency toward higher values in spring (Fig. 1e and f). Overall, the alpha-diversity analyses revealed consistent spatial gradients, alongside divergent temporal responses in both bacterial and fungal communities.

To quantify the drivers of microbial alpha diversity, GAMs were fitted separately for bacteria and fungi. The models explained 19.8% and 16.5% of the deviance in bacterial and fungal richness, respectively (bacteria: *adj. R*^2^ = 0.179; fungi: *adj. R*^2^ = 0.146, Fig. S4a and b). Spatial type was the dominant driver of microbial richness across both domains, accounting for the largest reduction in explained variance (bacteria: *ΔR*^2^ =0.077; fungi: *ΔR*^2^ =0.095), exceeding the sum of all temporal and seasonal components (bacteria *ΔR*^2^ = 0.020; fungi *ΔR*^2^ = 0.027). For long-term trends, no significant global trajectories were detected for either bacteria or fungi (both edf = 1.00, *P* > 0.05). While bacterial trend deviations across spatial types were non-significant (*P* > 0.05, Fig. S4a), fungal richness in underground environments exhibited a significant nonlinear temporal deviation (edf = 3.55, *F* = 3.62, $P\;=\;2.37\times 10^{-4}$, Fig. S4b), contributing the most to explained variance among all smooths (ΔR^2^ = 0.023 for fungi). Regarding seasonal dynamics, no significant global seasonal pattern emerged for either domain (Fig. S4c and d). However, bacterial richness in underground environments showed a significant seasonal deviation (edf = 3.20, *F* = 2.45, $P\;=\;5.37\times 10^{-4}$, Fig. S4c), reflecting complex within-year cyclical fluctuations unique to ventilation surface environments (*ΔR*^2^ = 0.003). No seasonal signals were detected for fungi in any spatial environment (all *P* > 0.05, Fig. S4d), indicating that fungal richness may not follow predictable within-year cycles in any of the sampled environments.

Beta-diversity analyses similarly revealed significant spatial structuring differences in both bacterial and fungal communities (PERMANOVA $R_{\text{bacteria}}^{2}\;=\;0.06$, $P_{\text{bacteria}}\;=\;0.001,\;\;R_{\text{fungi}}^{2}\;=\;0.06$, $P_{\text{fungi}}=\;0.001$, Fig. 1g and h). However, community dispersion differed significantly among spatial types in both domains (PERMDISP P=0.001 for both domains), suggesting that compositional variation could be driven by heterogeneous dispersion across different spaces. This was primarily attributable to the pronounced clustering of elevator samples relative to the broader scatter observed elsewhere, indicating strong environmental filtering within enclosed, human-managed habitats. Seasonal effect on fungal community composition was weak but significant (PERMANOVA $R^{2}\;=\;\text{0.04}$, P = 0.001; PERMDISP P = 0.6, Fig. 1j), whereas bacterial communities primarily varied in dispersion rather than centroid position (PERMANOVA $R^{2}\;=\;\text{0.04}$, P = 0.001; PERMDISP P = 0.001, Fig. 1i), highlighting distinct modes of temporal variability between the two domains.

Bacterial–fungal coupling closely tracked these spatial gradients (Fig. 1k). Across all samples, the two domains exhibited strong concordance (Procrustes r=0.84, P = 0.001; Mantel r=0.52, P = 0.001). Although significant alignment was detected within all individual spatial types (all P = 0.001), coupling strength decreased progressively from elevators (Procrustes r=0.982/0.186, SS=0.642; Mantel r=0.501) to aboveground (Procrustes r=0.907/0.420, SS=0.266; Mantel r=0.590), underground (Procrustes r=0.782/0.623, SS=0.311; Mantel r=0.505), and outdoor environments (Procrustes r=0.709/0.705, SS=0.239; Mantel r=0.731). Procrustes residuals differed significantly among spatial types (ANOVA, F=13.12, $P\;=\;3.26\times 10^{-8}$), with smaller residuals in enclosed environments reflecting tighter cross-domain congruence compared to the greater variability observed outdoors (Fig. S5).

Taken together, these findings reveal that spatial context overwhelmingly governs the dynamics of both bacterial and fungal communities at a localized scale. Enclosed environments, such as elevators, exhibited a distinct microbial profile: reduced beta-diversity suggests that physical shielding maintains stable community composition, while elevated alpha-diversity indicates a consistent influx of taxa from external sources, such as human occupants, jointly resulting in tightly clustered yet taxonomically rich assemblages with strong cross-domain coupling. While microclimatic variables and seasonal effects also contribute to community assembly, they tend to act as secondary modulators, ultimately constrained by the dominant spatial filtering.

### Spatiotemporal structure of pathogen-associated microbial communities

**Fig 2:**
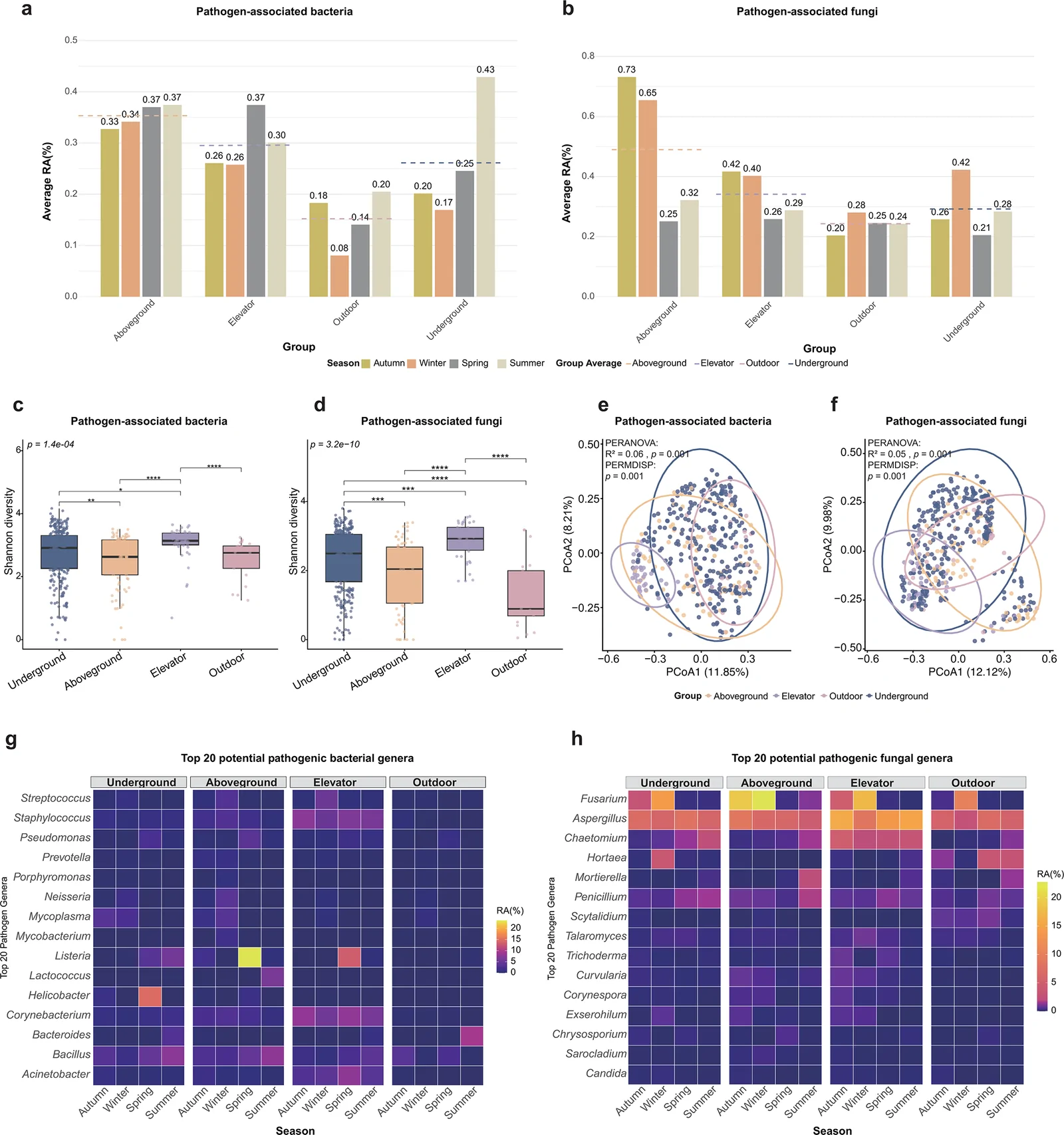
Spatial and seasonal variation in the diversity and composition of pathogen-associated microbial communities. (**a and b**) Summed relative abundance of pathogen-associated bacterial (**a**) and fungal (**b**) genera across spatial environments and seasons. Bars represent seasonal means within each spatial category; dashed lines indicate overall means per environment. (**c and d**) Shannon diversity of pathogen-associated bacterial (**c**) and fungal (**d**) communities across four spatial environments. (**e and f**) Principal coordinates analysis (PCoA) of pathogen-associated bacterial (**e**) and fungal (**f**) communities across spatial environments, with Bray–Curtis dissimilarity metric applied for both domains. (**g and h**) Heatmaps showing the relative abundance of the 20 most prevalent pathogen-containing bacterial (**g**) and fungal (**h**) genera across spatial environments and seasons.

Building upon the whole-community patterns, we next examined whether pathogen-associated taxa recapitulate these spatiotemporal rules or exhibit distinct ecological dynamics. Our results showed that pathogen-associated fungal genera were, on average, consistently more abundant than their bacterial counterparts across all spatial environments; the former peaked in abundance during autumn and winter, whereas the latter peaked in spring and summer. When accounting for spatial groups, taxa with pathogenic potential in both domains exhibited a distinct, matching gradient in relative abundance: aboveground > elevators > underground > outdoor (Fig. 2a and b). The alpha diversity of pathogen-associated bacterial and fungal communities largely mirrored the patterns of the total microbial community ($P_{\text{bacteria}}=1.4\times 10^{-4}$, $P_{\text{fungi}}=3.23\times 10^{-10}$), with elevators consistently harboring the highest diversity of pathogen-containing genera across all seasons (Fig. 2c and d). This suggests that rather than acting as restrictive ecological filters, enclosed transit hubs such as elevators may function as reservoirs of diverse taxa with pathogenic potential. Furthermore, the seasonal dynamics of pathogen-associated taxa diversity generally tracked those of the total community (Fig. S6a and b). However, this temporal signature was significantly more pronounced in taxa with pathogenic potential than in the total fungal community, characterized by a sharp decline in winter and a prominent peak in spring (Fig. S6b).

Beta-diversity analyses similarly revealed significant spatial differentiation in both pathogen-associated bacterial and fungal communities (Fig. 2e and f, PERMANOVA $R_{\text{bacteria}}^{2}=0.06$, $P_{\text{bacteria}}=0.001$; $R_{\text{fungi}}^{2}=0.05$, $P_{\text{fungi}}=0.001$). Globally, this structure was driven by the relative homogeneity of elevator samples, in contrast to the strong dispersion effects observed across the other three environments (PERMDISP P=0.001 for both domains). Interestingly, when compared directly to the tight clustering of their respective total communities (Fig. 1g and h), the pathogen-associated taxa within elevators showed increased heterogeneity. This structural decoupling suggests that localized micro-environmental dynamics and stochastic human inputs may introduce greater compositional volatility within the pathogenic fraction than in the background core microbiome. When all spatial groups were combined, clear seasonal partitioning emerged, particularly within the fungal domain (Fig. S6d, PERMANOVA $R_{\text{fungi}}^{2}=0.08$, $P_{\text{fungi}}=0.001$), where autumn and winter communities diverged distinctly from those in spring and summer. At the taxonomic level, this fungal macro-seasonal divergence was partially driven by the widespread enrichment of *Fusarium* across all spatial groups during the autumn and winter months (Fig. 2h). Conversely, while the seasonal signal was less apparent (PERMANOVA $R_{\text{bacteria}}^{2}=0.05$, $P_{\text{bacteria}}=0.001$) in the pathogen-associated bacterial community (Fig. S6c), prominent temporal dynamics emerged at the taxon level within specific spatial contexts. This localized variation was highlighted by the pronounced enrichment of *Listeria* in aboveground and elevator spaces, in contrast to the preferential enrichment of *Helicobacter* in underground environments in spring (Fig. 2g).

### Cross-domain ecological networks among pathogen-associated taxa across seasons

**Fig 3:**
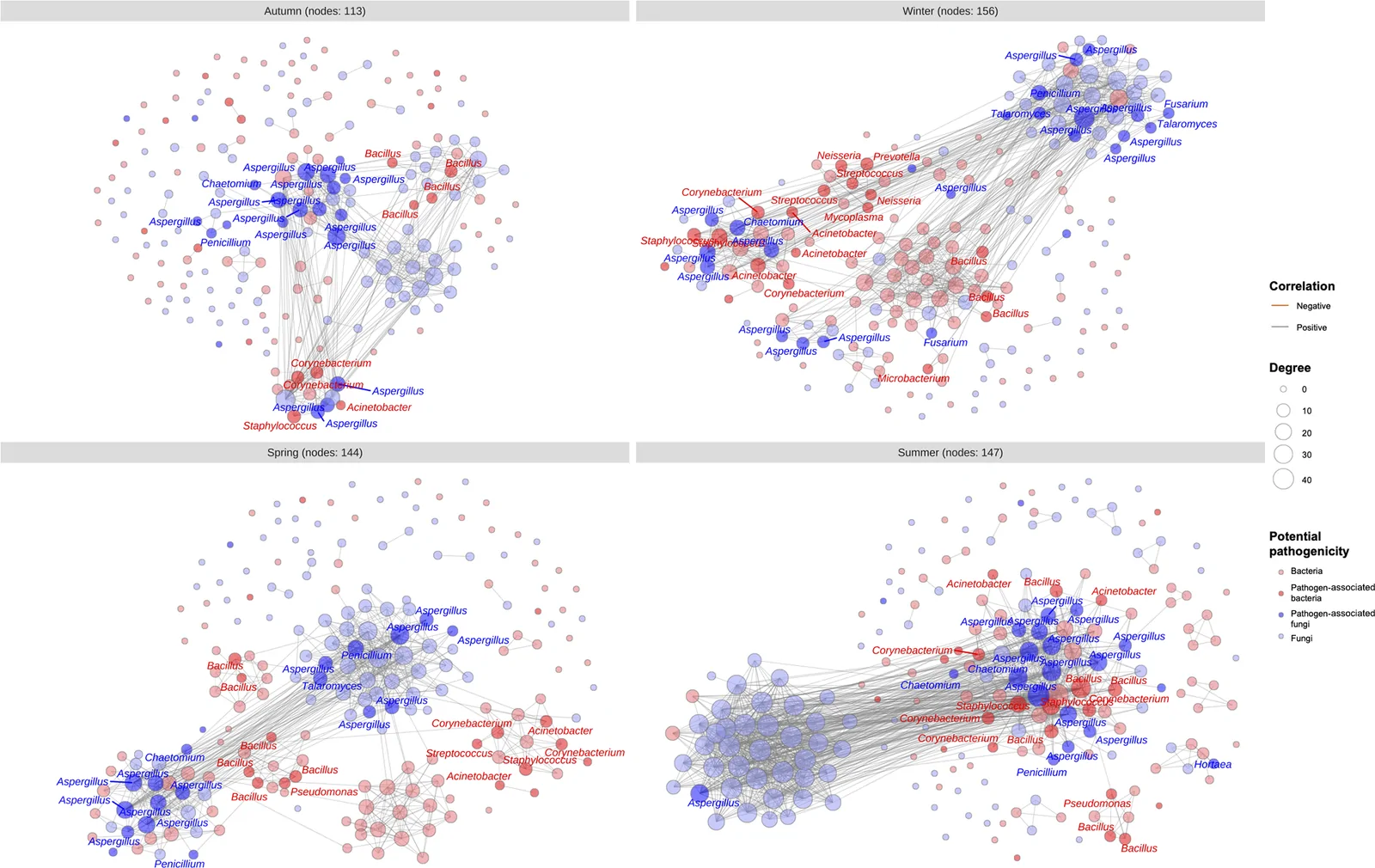
Cross-domain ecological networks of airborne microbial communities across seasons. Networks of inferred associations between bacterial and fungal ASVs were constructed using SPIEC-EASI. Only the top 200 most abundant bacterial and fungal ASVs were retained for network analysis. Each node represents an ASV and is colored by domain (bacteria or fungi) and potential pathogenicity status of the corresponding genus. Each edge indicates a significant association with an absolute coefficient greater than 0.6, and the color denotes the sign of the correlations. The network reveals extensive cross-domain associations, suggesting potential co-occurrence and exclusion patterns among airborne microbes.

To understand the associations between microbial taxa, bacterial and fungal genera were integrated to construct a cross-domain network for each season (Fig. 3). Our results revealed clear variations in network robustness across four seasons (Fig. S7). While natural connectivity declined year-round under targeted node removal, spring networks showed greater tolerance to the loss of highly connected nodes, and summer networks resisted the removal of bridging taxa. Strikingly, winter networks consistently exhibited the lowest robustness and most rapid decline in connectivity, suggesting that microbial associations were more vulnerable during the cold season.

Co-association patterns revealed dense, predominantly positive associations among potential pathogenic bacterial and fungal genera. The allergic fungal genus *Aspergillus*, which dominated across all the samples (Fig. 2h), consistently occupied a central hub position in the seasonal networks and maintained stable positive associations with another fungal genus, *Chaetomium*, throughout the year. Seasonal restructuring of association patterns was also evident: in autumn, *Aspergillus* showed positive associations with multiple bacterial and fungal genera, including *Corynebacterium*, *Staphylococcus*, and *Penicillium*. During winter, this hub expanded to encompass additional members, including *Talaromyces*, *Fusarium*, and *Acinetobacter*. A consistent positive association between fungal genera *Talaromyces* and *Penicillium* was also observed, reflecting coordinated dynamics among cold-favored fungi. Interdomain associations extended beyond *Aspergillus*; for example, *Chaetomium* showed positive correlations with the bacterial genera *Acinetobacter* and *Bacillus*. In spring, bacterial pathogen-containing genera formed tightly interconnected subclusters, in which *Staphylococcus* exhibited strong positive associations with *Acinetobacter*, *Bacillus*, *Corynebacterium*, and *Streptococcus*, indicating shared ecological niches or synchronous responses to environmental drivers. In summer, the network shifted toward a more bacteria-dominated structure with reduced intrafungal connectivity. In addition to the *Staphylococcus*-centered associations observed in spring, new positive correlations emerged between *Bacillus* and *Pseudomonas*, and between *Acinetobacter* and *Corynebacterium*, highlighting a seasonal reorganization of bacterial co-occurrence patterns under warm-season conditions.

### Climatic drivers of pathogen-containing genus distribution

Beyond the strong influence of spatial context, seasonal shifts in environmental conditions (i.e., relative humidity and temperature) exerted a secondary but crucial influence on community structure. At the community level, the richness of both bacterial and fungal communities exhibited a weak but significant negative correlation with relative humidity and temperature, indicating a general suppression under warmer, more humid conditions (Fig. S8). However, the richness of both domains peaked at a relative humidity of 50%–60% and a temperature of 15°C–17°C, a pattern more pronounced in fungi, suggesting conditions favored by the majority of microbes.

**Fig 4:**
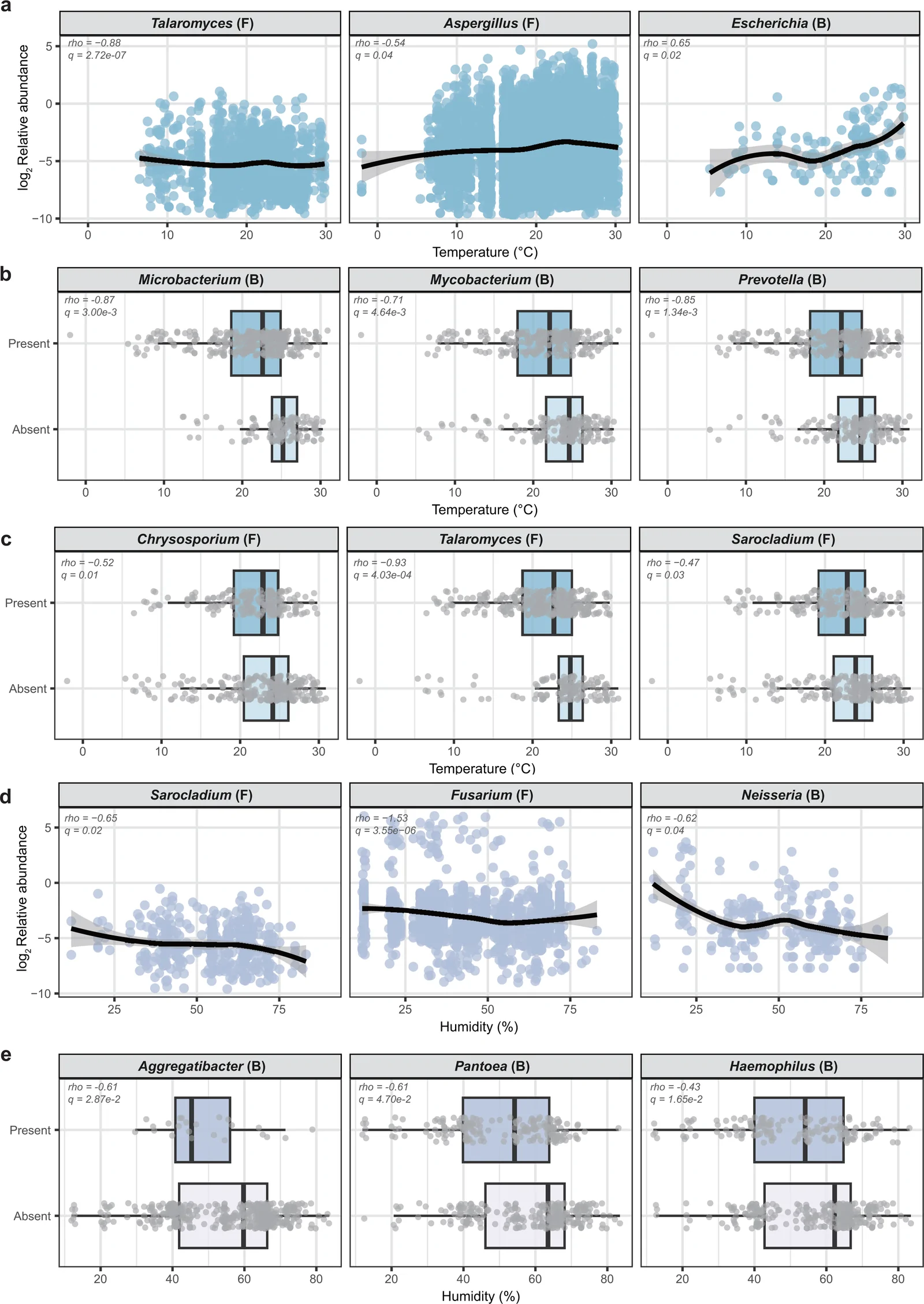
Environmental drivers of the dynamics of the pathogen-containing bacterial and fungal genera. (**a**) Correlations between temperature and the log₂-transformed relative abundance of pathogen-containing bacterial and fungal genera, including *Escherichia*, *Talaromyces*, and *Aspergillus*. (**b and c**) Comparison of temperature between samples with and without detection of pathogen-containing bacterial (*Microbacterium*, *Mycobacterium*, and *Prevotella*) (**b**) and fungal genera (*Chrysosporium*, *Talaromyces*, and *Sarocladium*) (**c**). (**d**) Correlations between relative humidity and log₂-transformed relative abundance of pathogen-containing bacterial and fungal genera (*Neisseria*, *Sarocladium*, and *Fusarium*). (**e**) Comparison of relative humidity between samples with and without detection of pathogen-containing bacterial genera (*Aggregatibacter*, *Pantoea*, and *Haemophilus*). The labels B and F in the parentheses denote the respective microbial domain: B, bacteria; F, fungi.

The responses of individual taxa, especially those with pathogenic potential, to the changes in environmental conditions were further assessed using MaAsLin3 (Table S1). Temperature exhibited a taxon-dependent influence. In terms of relative abundance, pathogen-associated fungal genera such as *Talaromyces* and *Aspergillus* declined as temperature increased, whereas a positive association was found in the pathogen-containing bacterial genus *Escherichia* (Fig. 4a). When considering only the presence/absence of taxa, several potential pathogenic bacterial genera, including *Microbacterium*, *Mycobacterium*, and *Prevotella*, showed decreasing prevalence with increasing temperature (Fig. 4b), indicating enhanced persistence under cooler conditions. A similar pattern was also found in the pathogen-containing fungal genera, including *Talaromyces*, *Sarocladium*, and *Chrysosporium* (Fig. 4c).

Unlike temperature, relative humidity showed a predominantly negative influence on both the prevalence and relative abundance of pathogen-containing genera. *Neisseria*, together with potential fungal pathogens such as *Sarocladium* and *Fusarium*, exhibited marked declines in relative abundance as relative humidity increased (Fig. 4d), suggesting a preference for drier environments consistent with their elevated winter representation (Fig. 2g and h). Likewise, several potential bacterial pathogens, including *Aggregatibacter*, *Pantoea*, and *Haemophilus*, were more prevalent under less humid conditions (Fig. 4e). Collectively, these results indicate that relative humidity and temperature act as key micro-environmental filters that shape pathogen-associated genus distributions in a highly taxon-specific manner.

### Seasonal modulation of bacterial transcriptional activity

**Fig 5:**
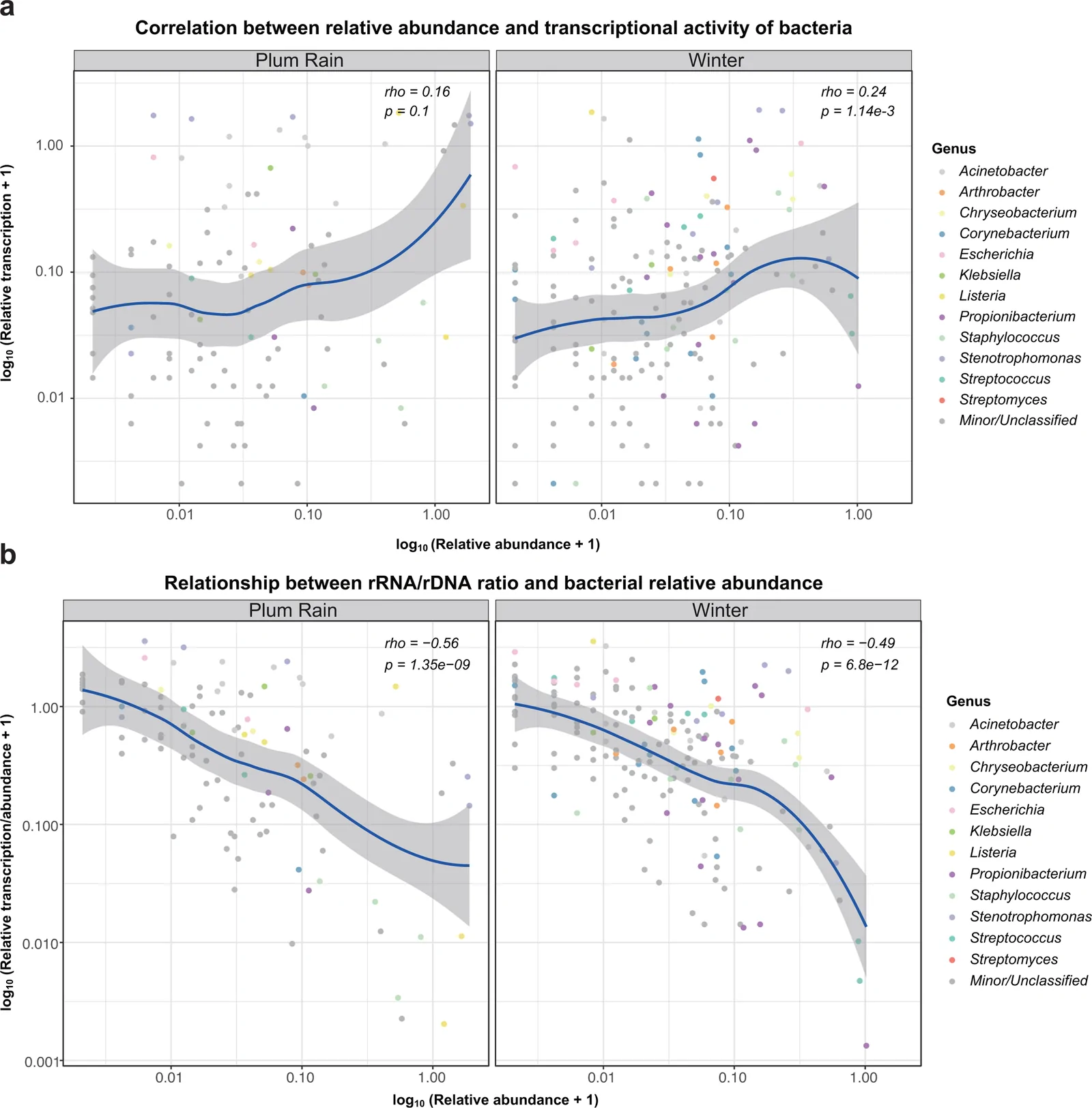
Transcriptional activity of airborne bacterial taxa in underground environments. (**a**) Spearman correlations between relative abundance (rDNA) and relative transcriptional activity (rRNA) of bacterial ASVs in winter and plum rain summer season samples. (**b**) Relationships between rRNA/rDNA ratio and relative abundance of bacterial ASVs across the same conditions. Each point represents an ASV detected in both rRNA and rDNA pools from the parallel samples. The blue line indicates the fitted linear regression trend. ASVs are taxonomically annotated at the genus level. A ratio >1 indicates putative metabolically active taxa, whereas values between 0 and 1 indicate low or inactive transcriptional states.

Given the significant climatic filtering observed, we further examined the influence of environmental conditions on bacterial transcriptional activity by comparing rRNA-based (active) and rDNA-based (total) sequencing data from two climatically contrasting seasons: winter and the summer plum rain period. A weak but positive correlation between the relative abundance of bacterial members in the active and total community pools was observed in winter (Spearman’s ρ = 0.24, *P* < 0.001). In contrast, a similar trend was observed, but not statistically significant, during the plum rain period (Spearman’s ρ = 0.16, *P* = 0.1; Fig. 5a).

To rigorously investigate this relationship, we quantified the relative transcriptional activity of individual bacterial ASVs using the rRNA/rDNA ratio, where higher values are interpreted as a proxy for increased transcriptional activity. In both seasons, ASVs with higher relative abundances in the total community generally exhibited lower transcriptional activity (Spearman’s $\rho_{plum\_rain}\;=\;-0.56,\;\;\rho_{winter}\text{=}-\text{0.49},P\;<\;0.001$ for both seasons; Fig. 5b), indicating that dominance in the total community does not necessarily translate to heightened metabolic vigor. Nonetheless, taxon-specific transcriptional dynamics were evident among co-detected pathogen-associated ASVs. Certain ASVs from *Acinetobacter*, *Escherichia*, and *Stenotrophomonas* maintained consistently high relative activity in both seasons. Pronounced seasonal contrasts also emerged: a *Klebsiella* ASV displayed enhanced transcriptional activity in summer, while ASVs from *Propionibacterium*, *Corynebacterium*, *Listeria*, and *Streptococcus* were metabolically more active in winter. These season-dependent bacterial transcriptional profiles complement the abundance dynamics and climatic correlations documented earlier, reinforcing the concept that environmental conditions not only reshape community composition but also actively modulate the metabolic state of persistent microbes with pathogenic potential.

### Dispersal dynamics and cross-spatial distribution of pathogen-associated taxa

**Fig 6:**
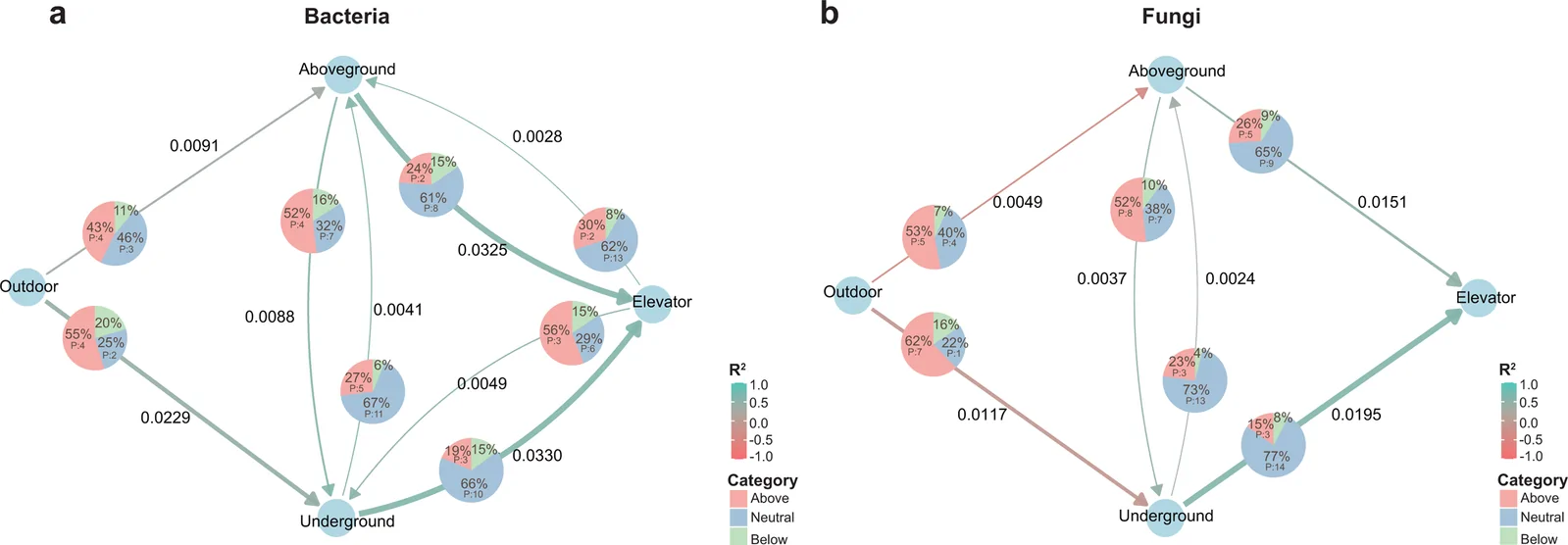
Inferred diffusion routes of airborne bacteria and fungi across four spatial environments. (**a and b**) Inferred dispersal pathways of bacterial (**a**) and fungal (**b**) communities among spatial environments within the buildings. Directional arrows indicate dispersal routes, with arrow thickness proportional to the coefficient of determination (*R*^2^), reflecting the strength of each pathway. The decimals adjacent to the arrows represent migration rates (M), where higher values denote greater dispersal potential. The embedded pie charts illustrate the proportions of ASVs distributed across the above-neutral, neutral, and below-neutral partitions; the label P within the above-neutral and neutral partitions indicates the specific counts of pathogen-containing genera unique to those environments.

To determine the extent to which dispersal processes contribute to the observed spatial patterns across different environments, we evaluated cross-spatial taxon sharing. A substantial core microbiota—comprising 53% of bacterial and 36% of fungal taxa—was ubiquitous across all four spatial settings (Fig. S9). Crucially, this shared pool encompassed 35 and 37 pathogen-containing bacterial and fungal genera, respectively, highlighting the pervasive baseline distribution of clinically relevant microorganisms across complex BEs. We then quantified the dispersal pattern of these potential pathogens between outdoor and indoor environments, and among distinct indoor spatial compartments using the Sloan NCM. Overall, both bacterial and fungal communities exhibited relatively low migration rates, though bacterial taxa showed slightly higher dispersal potential than fungal taxa (Fig. 6). Among all modeled dispersal routes, migration from aboveground and underground environments toward elevators consistently exhibited the highest rates for both domains. This suggests that elevators may serve as important nodes for cross-spatial microbial exchange, a finding structurally congruent with their elevated diversity and tight bacterial–fungal coupling established in our previous analyses (Fig. 1k).

Despite generally constrained macro-level dispersal, specific pathogen-associated taxa exhibited potent migration capacities (Table S2). Within the fungal domain, *Aspergillus* and *Candida* consistently fell into the above-neutral category across all dispersal pathways, indicating elevated occurrence frequencies and enhanced dispersal potential. This strongly corroborates the ecological ubiquity and persistent abundance of *Aspergillus* observed throughout our study. *Alternaria* and *Talaromyces* also showed elevated dispersal signals across all four routes, while *Purpureocillium*, *Penicillium*, *Trichosporon*, and *Curvularia* exhibited above-neutral distribution in at least two pathways. Within the bacterial domain, *Acinetobacter*, *Streptococcus*, *Prevotella*, and *Clostridium* exhibited above-neutral dispersal across all four routes. This highlights their strong dispersal capabilities, which directly support their broad spatial representation and central roles within the cross-domain networks. Ultimately, these findings establish that although community-level dispersal was generally constrained, specific taxa with pathogenic potential exhibited disproportionately high migration potential, indicating that dispersal processes operate heterogeneously across distinct taxa.

## DISCUSSION

BEs constitute the primary setting of human microbial exposure, yet the ecological mechanisms governing the distribution and persistence of potentially pathogenic microorganisms within these systems remain incompletely understood. By integrating year-round observations across multiple spatial environments with community analyses and ecological modeling, this study revealed that pathogen-associated microbial communities were not randomly assembled but were structured by the combined effects of spatial filtering, seasonal forcing, microbial associations, and dispersal. These processes operated in a coordinated manner, forming an interconnected ecological system that regulates the occurrence, persistence, and dispersal across space and time.

Among these processes, spatial filtering establishes the primary structure of pathogen-associated communities. Consistent with patterns observed in total microbial assemblages, both the diversity and composition of pathogen-associated subsets varied strongly across spatial environments, with enclosed indoor spaces exerting the strongest selective pressures. Notably, elevators exhibited a distinct ecological signature characterized by simultaneously elevated diversity and limited community heterogeneity of potential pathogens. This pattern reflects the interplay between continuous microbial input and deterministic environmental filtering. While frequent human-associated inputs introduce diverse taxa, restricted airflow and confinement create relatively stable conditions that impose persistent yet monotonous selection, leading to a highly diversified microbial community co-occurring with reduced compositional variability (1). Similar dynamics have been reported in other enclosed, high-density environments, such as hospital wards, subway systems, and aircraft cabins, where architectural confinement and sustained human traffic collectively promote the accumulation and persistence of opportunistic pathogens, thereby establishing these spaces as reservoirs with heightened potential for pathogen transmission (23–25). Our results extend this framework by showing that elevators function not only as accumulation sites but also as selective environments that stabilize potential microbial pathogens, amplifying their ecological and epidemiological importance within buildings.

The strong spatial filtering resulted in overall low migration rates of microorganisms across different spatial environments; nevertheless, several key taxa exhibited effective cross-space distribution, possibly via human-mediated and airflow vectors (26). The spore-forming, pathogen-containing fungal genera, such as *Aspergillus* and *Candida*, were particularly prominent, reflecting their efficient dissemination and potential airborne transmission via small, resilient spores (27, 28). Similarly, several pathogen-containing bacterial genera, including *Acinetobacter*, *Streptococcus*, *Prevotella*, and *Clostridium*, also exhibited broad distribution across multiple spatial compartments. Among these, human-associated taxa such as *Streptococcus* and *Prevotella* are likely redistributed through respiratory bioaerosols and skin shedding (29, 30). In contrast, desiccation-tolerant environmental genera such as *Acinetobacter* and *Clostridium* may persist on inanimate surfaces, such as ventilation filters, under drier conditions and be secondary aerosolized and further dispersed via disturbance-driven dust resuspension (31, 32).

Superimposed on this spatial baseline, seasonal climatic variation further modulates the dynamics of potential microbial pathogens, but with markedly different effects between bacteria and fungi. Potential fungal pathogens exhibited pronounced seasonal shifts, with elevated relative abundance across four spatial environments during autumn and winter. This pattern does not necessarily reflect optimal fungal growth conditions, as most indoor fungi thrive at moderate temperatures (typically 20°C–30°C) and high relative humidity (>70%), as previously reported (33). Instead, this phenomenon may result from a combination of increased spore persistence, reduced microbial competition, and enhanced aerosolization under low-humidity conditions rather than *in situ* growth dynamics (33, 34). For instance, the spore-producing fungal genus *Fusarium* generally prefers moist environments for active growth (35); thus, its increased relative abundance during autumn and winter likely reflects enhanced environmental loading from plant-derived sources (36), combined with improved aerosolization under drier atmospheric conditions (37), thereby increasing suspension time and transport efficiency. Concurrently, the reduced activity of other competing taxa during colder periods may create ecological opportunities that allow *Fusarium* to persist, even if its intrinsic growth rate is suboptimal.

The temporal dynamics of potential bacterial pathogens appeared to be governed by taxon-specific physiological strategies rather than uniform seasonal responses. Although *Listeria* exhibited lower relative abundance during winter, its transcriptional activity was paradoxically higher than in summer. Rather than contradicting the capacity of potential bacterial pathogens to persist under cold stress, this pattern is consistent with the well-established cold-adaptation strategy of *Listeria* species such as *L. monocytogenes*, which actively upregulates cold shock proteins and stress-associated sigma factors at low temperatures to maintain cellular viability (38). The elevated transcriptional activity observed in winter, therefore, likely reflects active stress adaptation rather than metabolic dormancy. With the transition into spring, the relaxation of cold-induced stress, together with moderate increases in temperature and relative humidity, may shift cellular resource allocation from stress tolerance toward growth and replication, thereby promoting population expansion and the marked increase in relative abundance observed during this period. Simultaneously, the seasonal decline in fungal dominance may partially alleviate interkingdom competitive constraints, thereby creating ecological space conducive to the proliferation of potential bacterial pathogens. These asynchronous seasonal dynamics between two domains indicate that pathogen risk does not diminish uniformly throughout the year but instead shifts in composition, with potential fungal pathogens dominating under colder conditions and bacterial counterparts becoming more prominent during transitional and warmer periods.

Beyond spatial and environmental constraints, the association between microbes may also play a critical role in shaping the persistence of potential pathogens. Cross-domain network analyses revealed that pathogen-associated taxa are embedded in densely connected, predominantly positive-association networks. These consistent, robust co-occurrence patterns may reflect shared ecological preferences, localized environmental filtering, or potential mutually beneficial associations. Notably, the allergic fungal genus *Aspergillus* persistently occupied a central hub position across all seasons. While its keystone role may be attributed to a combined strategy of ecological versatility, desiccation tolerance, and continuous dispersal via airborne spores (7, 28), its network centrality suggests that it may act as a physical or structural anchor for other taxa. In generally dry BEs, dense fungal structures and spore accumulations can alter surface topography, creating micro-niches that retain trace moisture and organic matter, thereby potentially facilitating the attachment and survival of other fungal and bacterial partners (39–41). This combination of high physical tenacity and localized niche stabilization may plausibly contribute to the strong, positive cross-domain coupling centered on *Aspergillus*, ultimately enhancing the collective persistence of potentially pathogenic taxa within the indoor microbiota.

Crucially, the seasonal variation in network topology indicates that the associations between microbes are dynamically reorganized rather than statically maintained. Although our structural analyses showed that winter networks were more vulnerable to targeted node removal, they did not collapse; rather, they shifted toward a heavily fungus-dominated configuration with sustained cross-domain associations. This structural reorganization is likely driven by the seasonal enrichment of the dominating genus *Fusarium* and the less common genus *Talaromyces*. In contrast, the dominance of pathogen-containing bacterial genera in the summer network, including *Bacillus*, *Staphylococcus*, and *Corynebacterium*, was accompanied by reduced intrafungal connectivity. These seasonal transitions suggest that microbial association networks are shaped by distinct ecological strategies across climatic conditions: winter communities appear to be maintained largely by the persistence and dispersal of fungal spores, whereas summer communities are characterized by enhanced bacterial activity and intensified co-association patterns. Collectively, these findings demonstrate that seasonal dynamics reshape not only community composition but also the fundamental balance and interaction architecture of potential pathogen networks.

Taken together, our study supports a unified framework in which the dynamics of pathogen-associated taxa in BEs emerge from the coupled effects of spatial filtering, climatic modulation, and microbial associations. Within this system, elevated migration potential may pave the initial colonization of keystone taxa, while subsequent cross- and intra-domain associations further promote their long-term surface persistence. Elevators emerge as critical nodes within this framework; the stable environmental conditions they provide, coupled with relatively high rates of inbound migration, position them as primary reservoirs for potential pathogens. Because these ecological processes are intrinsically linked, they jointly govern the persistence and redistribution of potential pathogens across the building. This perspective has clear, actionable implications for future intervention strategies. Approaches targeting a single factor, such as uniform disinfection or static environmental control, are less effective in disrupting this coordinated system. More effective management should prioritize structurally critical nodes, such as the ventilation interfaces within elevators, while dynamically accounting for seasonal shifts in dominant pathogen groups and the stabilizing roles of keystone taxa. Looking forward, as climate change reshapes indoor temperature and humidity regimes, progress toward predictive opportunistic pathogen control will depend heavily on integrating microbial activity profiling with high-resolution micro-environmental monitoring and airflow-informed architectural modeling.

These conclusions should be interpreted in light of several limitations. Amplicon-based sequencing optimizes broad community profiling but restricts taxonomic resolution to the genus level, limiting the precise identification of pathogenic species and their virulence traits. Furthermore, this pathogenic genus-level assignment may overestimate pathogen prevalence by including nonpathogenic members within broadly defined taxa. Transcriptional activity was assessed primarily for bacterial communities and across two seasons, providing an initial framework that may not fully capture the full extent of functional dynamics, particularly for fungi. In addition, rRNA-based metrics provide a valuable proxy for estimating transcriptional activity, though they may be influenced by variation in ribosomal copy number and physiological state. Finally, neutral modeling and co-occurrence networks can infer potential dispersal and associations, but they do not establish strict causality. Future work integrating metagenomics, metatranscriptomics, and quantitative airflow modeling will be essential for further elucidating these mechanisms and improving the prediction of pathogen dynamics in BEs.

## Data Availability

The raw DNA and RNA sequencing data for bacterial and fungal communities have been deposited in the NCBI Sequence Read Archive (SRA) database under the BioProject accession numbers PRJNA1268906 and PRJNA1466084, respectively. The metadata and in-house scripts for data analysis are available at https://github.com/Shijiao7/XJTLU_Air_Microbiome.

## SUPPLEMENTAL MATERIAL

Supplemental figures spectrum.01915-26-s0001.pdf Fig. S1 to S9.

Tables S1 and S2 spectrum.01915-26-s0002.xlsx Results related to MaAsLin3 and Sloan Neutral Model analyses.

ASM does not own the copyrights to Supplemental Material that may be linked to, or accessed through, an article. The authors have granted ASM a non-exclusive, world-wide license to publish the Supplemental Material files. Please contact the corresponding author directly for reuse.

## References

[1] Leung MH, Tong X, Lee PK. 2019. Indoor microbiome and airborne pathogens. Compr Biotechnol 96. doi:10.1016/B978-0-444-64046-8.00477-8

[2] Šunić I, Šarac J, Havaš Auguštin D, Pozdniakova S, Ferguson RMW, Jergović M, Visentin D, Borràs S, Archer E, Henderson DK, Vitko S, Ašić A, Bošnjaković A, Maglica Ž, Viegas C, Novokmet N, Karlović N, Marjanović D, Muszyński A, Liu Y, Karisola P, Alenius H, Krych L, Lovrić M. 2026. The indoor microbiome: sampling, analysis and emerging trends. Environ Microbiol Rep 18:e70272. doi:10.1111/1758-2229.70272 PMC13054241 41943876

[3] Adams RI, Bateman AC, Bik HM, Meadow JF. 2015. Microbiota of the indoor environment: a meta-analysis. Microbiome 3:49. doi:10.1186/s40168-015-0108-3 PMC4604073 26459172

[4] Dong X, Wu Y, Chen X, Li H, Cao B, Zhang X, Yan X, Li Z, Long Y, Li X. 2021. Effect of thermal, acoustic, and lighting environment in underground space on human comfort and work efficiency: a review. Sci Total Environ 786:147537. doi:10.1016/j.scitotenv.2021.147537

[5] Li X, Feng B. 2023. Transmission of droplet aerosols in an elevator cabin: effect of the ventilation mode. Build Environ 236:110261. doi:10.1016/j.buildenv.2023.110261 PMC10066590 37041765

[6] Chen Y, Liang Z, Li G, An T. 2024. Indoor/Outdoor airborne microbiome characteristics in residential areas across four seasons and its indoor purification. Environ Int 190:108857. doi:10.1016/j.envint.2024.108857 38954924

[7] Zhu N, Xu J, Li K, Luo Y, Yang D, Zhou C. 2019. Spatiotemporal change of plum rains in the Yangtze River Delta and its relation with EASM, ENSO, and PDO during the period of 1960–2012. Atmosphere (Basel) 10:258. doi:10.3390/atmos10050258

[8] Zhou Y, Leung MHY, Tong X, Lai Y, Tong JCK, Ridley IA, Lee PKH. 2021. Profiling airborne microbiota in mechanically ventilated buildings across seasons in Hong Kong reveals higher metabolic activity in low-abundance bacteria. Environ Sci Technol 55:249–259. doi:10.1021/acs.est.0c06201 33346641

[9] Bolyen E, Rideout JR, Dillon MR, Bokulich NA, Abnet CC, Al-Ghalith GA, Alexander H, Alm EJ, Arumugam M, Asnicar F, et al.. 2019. Reproducible, interactive, scalable and extensible microbiome data science using QIIME 2. Nat Biotechnol 37:852–857. doi:10.1038/s41587-019-0209-9 PMC7015180 31341288

[10] Callahan BJ, McMurdie PJ, Rosen MJ, Han AW, Johnson AJA, Holmes SP. 2016. DADA2: high-resolution sample inference from Illumina amplicon data. Nat Methods 13:581–583. doi:10.1038/nmeth.3869 PMC4927377 27214047

[11] McDonald D, Jiang Y, Balaban M, Cantrell K, Zhu Q, Gonzalez A, Morton JT, Nicolaou G, Parks DH, Karst SM, Albertsen M, Hugenholtz P, DeSantis T, Song SJ, Bartko A, Havulinna AS, Jousilahti P, Cheng S, Inouye M, Niiranen T, Jain M, Salomaa V, Lahti L, Mirarab S, Knight R. 2024. Greengenes2 unifies microbial data in a single reference tree. Nat Biotechnol 42:715–718. doi:10.1038/s41587-023-01845-1 PMC10818020 37500913

[12] Nilsson RH, Larsson K-H, Taylor AFS, Bengtsson-Palme J, Jeppesen TS, Schigel D, Kennedy P, Picard K, Glöckner FO, Tedersoo L, Saar I, Kõljalg U, Abarenkov K. 2019. The UNITE database for molecular identification of fungi: handling dark taxa and parallel taxonomic classifications. Nucleic Acids Res 47:D259–D264. doi:10.1093/nar/gky1022 PMC6324048 30371820

[13] McMurdie PJ, Holmes S. 2013. Phyloseq: an R package for reproducible interactive analysis and graphics of microbiome census data. PLoS One 8:e61217. doi:10.1371/journal.pone.0061217 PMC3632530 23630581

[14] Anderson MJ, Walsh DCI. 2013. PERMANOVA, ANOSIM, and the Mantel test in the face of heterogeneous dispersions: what null hypothesis are you testing? Ecol Monogr 83:557–574. doi:10.1890/12-2010.1

[15] Anderson MJ. 2006. Distance-based tests for homogeneity of multivariate dispersions. Biometrics 62:245–253. doi:10.1111/j.1541-0420.2005.00440.x 16542252

[16] Guo C, Chen Q, Fan G, Sun Y, Nie J, Shen Z, Meng Z, Zhou Y, Li S, Wang S, Ma J, Sun Q, Wu L. 2024. gcPathogen: a comprehensive genomic resource of human pathogens for public health. Nucleic Acids Res 52:D714–D723. doi:10.1093/nar/gkad875 PMC10767814 37850635

[17] Meechan PJ, Potts J. 2020. Biosafety in microbiological and biomedical laboratories.

[18] Johnson JS, Spakowicz DJ, Hong B-Y, Petersen LM, Demkowicz P, Chen L, Leopold SR, Hanson BM, Agresta HO, Gerstein M, Sodergren E, Weinstock GM. 2019. Evaluation of 16S rRNA gene sequencing for species and strain-level microbiome analysis. Nat Commun 10:5029. doi:10.1038/s41467-019-13036-1 PMC6834636 31695033

[19] Nickols WA, Kuntz T, Shen J, Maharjan S, Mallick H, Franzosa EA, Thompson KN, Nearing JT, Huttenhower C. 2026. MaAsLin 3: refining and extending generalized multivariable linear models for meta-omic association discovery. Nat Methods 23:554–564. doi:10.1038/s41592-025-02923-9 PMC12982127 41540124

[20] Sloan WT, Lunn M, Woodcock S, Head IM, Nee S, Curtis TP. 2006. Quantifying the roles of immigration and chance in shaping prokaryote community structure. Environ Microbiol 8:732–740. doi:10.1111/j.1462-2920.2005.00956.x 16584484

[21] Burns AR, Stephens WZ, Stagaman K, Wong S, Rawls JF, Guillemin K, Bohannan BJ. 2016. Contribution of neutral processes to the assembly of gut microbial communities in the zebrafish over host development. ISME J 10:655–664. doi:10.1038/ismej.2015.142 PMC4817674 26296066

[22] Kurtz ZD, Müller CL, Miraldi ER, Littman DR, Blaser MJ, Bonneau RA. 2015. Sparse and compositionally robust inference of microbial ecological networks. PLoS Comput Biol 11:e1004226. doi:10.1371/journal.pcbi.1004226 PMC4423992 25950956

[23] Argyropoulos CD, Skoulou V, Efthimiou G, Michopoulos AK. 2023. Airborne transmission of biological agents within the indoor built environment: a multidisciplinary review. Air Qual Atmos Health 16:477–533. doi:10.1007/s11869-022-01286-w PMC9703444 36467894

[24] Gohli J, Bøifot KO, Moen LV, Pastuszek P, Skogan G, Udekwu KI, Dybwad M. 2019. The subway microbiome: seasonal dynamics and direct comparison of air and surface bacterial communities. Microbiome 7:160. doi:10.1186/s40168-019-0772-9 PMC6924074 31856911

[25] Osman S, La Duc MT, Dekas A, Newcombe D, Venkateswaran K. 2008. Microbial burden and diversity of commercial airline cabin air during short and long durations of travel. ISME J 2:482–497. doi:10.1038/ismej.2008.11 PMC7099242 18256704

[26] Gilbert JA, Stephens B. 2018. Microbiology of the built environment. Nat Rev Microbiol 16:661–670. doi:10.1038/s41579-018-0065-5 30127345

[27] Mousavi B, Hedayati MT, Hedayati N, Ilkit M, Syedmousavi S. 2016. *Aspergillus* species in indoor environments and their possible occupational and public health hazards. Curr Med Mycol 2:36–42. doi:10.18869/acadpub.cmm.2.1.36 PMC5490296 28681011

[28] Paulussen C, Hallsworth JE, Álvarez-Pérez S, Nierman WC, Hamill PG, Blain D, Rediers H, Lievens B. 2017. Ecology of aspergillosis: insights into the pathogenic potency of *Aspergillus fumigatus* and some other *Aspergillus* species. Microb Biotechnol 10:296–322. doi:10.1111/1751-7915.12367 PMC5328810 27273822

[29] Nazaroff WW. 2016. Indoor bioaerosol dynamics. Indoor Air 26:61–78. doi:10.1111/ina.12174 PMC7165847 25483392

[30] Hospodsky D, Qian J, Nazaroff WW, Yamamoto N, Bibby K, Rismani-Yazdi H, Peccia J. 2012. Human occupancy as a source of indoor airborne bacteria. PLoS One 7:e34867. doi:10.1371/journal.pone.0034867 PMC3329548 22529946

[31] Farrow JM, Wells G, Pesci EC. 2018. Desiccation tolerance in *Acinetobacter baumannii* is mediated by the two-component response regulator BfmR. PLoS One 13:e0205638. doi:10.1371/journal.pone.0205638 PMC6181384 30308034

[32] Knight DR, Elliott B, Chang BJ, Perkins TT, Riley TV. 2015. Diversity and evolution in the genome of *Clostridium difficile*. Clin Microbiol Rev 28:721–741. doi:10.1128/CMR.00127-14 PMC4475645 26085550

[33] Segers FJJ, van Laarhoven KA, Huinink HP, Adan OCG, Wösten HAB, Dijksterhuis J. 2016. The indoor fungus *Cladosporium halotolerans* survives humidity dynamics markedly better than *Aspergillus niger* and penicillium rubens despite less growth at lowered steady-state water activity. Appl Environ Microbiol 82:5089–5098. doi:10.1128/AEM.00510-16 PMC4988216 27316968

[34] Segers FJJ, Dijksterhuis J, Giesbers M, Debets AJM. 2023. Natural folding of airborne fungal spores: a mechanism for dispersal and long-term survival? Fungal Biol Rev 44:100292. doi:10.1016/j.fbr.2022.10.005

[35] Chakrapani K, Chanu WT, Sinha B, Thangjam B, Hasan W, Devi KS, Chakma T, Phurailatpam S, Mishra LK, Singh GM, Khoyumthem P, Saini R. 2023. Deciphering growth abilities of *Fusarium oxysporum* f. sp. *pisi* under variable temperature, pH and nitrogen. Front Microbiol 14:1228442. doi:10.3389/fmicb.2023.1228442 PMC10435999 37601368

[36] Li Z, He F, Gai X, Zhu H, He S, Xuan YH, Bai L. 2025. The growth, development and infection process of the plant pathogen *Fusarium*. Plant Signal Behav 20:2573097. doi:10.1080/15592324.2025.2573097 PMC12582100 41176708

[37] Hoffmann A, Funk R, Müller MEH. 2021. Blowin’ in the wind: wind dispersal ability of phytopathogenic fusarium in a wind tunnel experiment. Atmosphere (Basel) 12:1653. doi:10.3390/atmos12121653

[38] Kim U, Oh S-W. 2025. Freeze-thaw resilience of *Shigella flexneri* NCCP 10852 in co-culture with *Listeria monocytogenes*: implications for biofilm-mediated survival in cold environment. Int J Food Microbiol 443:111402. doi:10.1016/j.ijfoodmicro.2025.111402 40882594

[39] Deveau A, Bonito G, Uehling J, Paoletti M, Becker M, Bindschedler S, Hacquard S, Hervé V, Labbé J, Lastovetsky OA, Mieszkin S, Millet LJ, Vajna B, Junier P, Bonfante P, Krom BP, Olsson S, van Elsas JD, Wick LY. 2018. Bacterial-fungal interactions: ecology, mechanisms and challenges. FEMS Microbiol Rev 42:335–352. doi:10.1093/femsre/fuy008 29471481

[40] Villa F, Cappitelli F. 2019. The ecology of subaerial biofilms in dry and inhospitable terrestrial environments. Microorganisms 7:380. doi:10.3390/microorganisms7100380 PMC6843906 31547498

[41] Worrich A, Stryhanyuk H, Musat N, König S, Banitz T, Centler F, Frank K, Thullner M, Harms H, Richnow HH, Miltner A, Kästner M, Wick LY. 2017. Mycelium-mediated transfer of water and nutrients stimulates bacterial activity in dry and oligotrophic environments. Nat Commun 8:15472. doi:10.1038/ncomms15472 PMC5467244 28589950

