# Supplementary material for: Architectural confinement and seasonal forcing shape cross-domain pathogen-associated assemblages in complex built environments
Source: Microbiol Spectr. 2026 Aug 31;14(10):e01915-26. doi: 10.1128/spectrum.01915-26 (PMC13689491; doi:10.1128/spectrum.01915-26)
Supplement: Supplementary file 1 [file spectrum.01915-26-s0001.pdf]

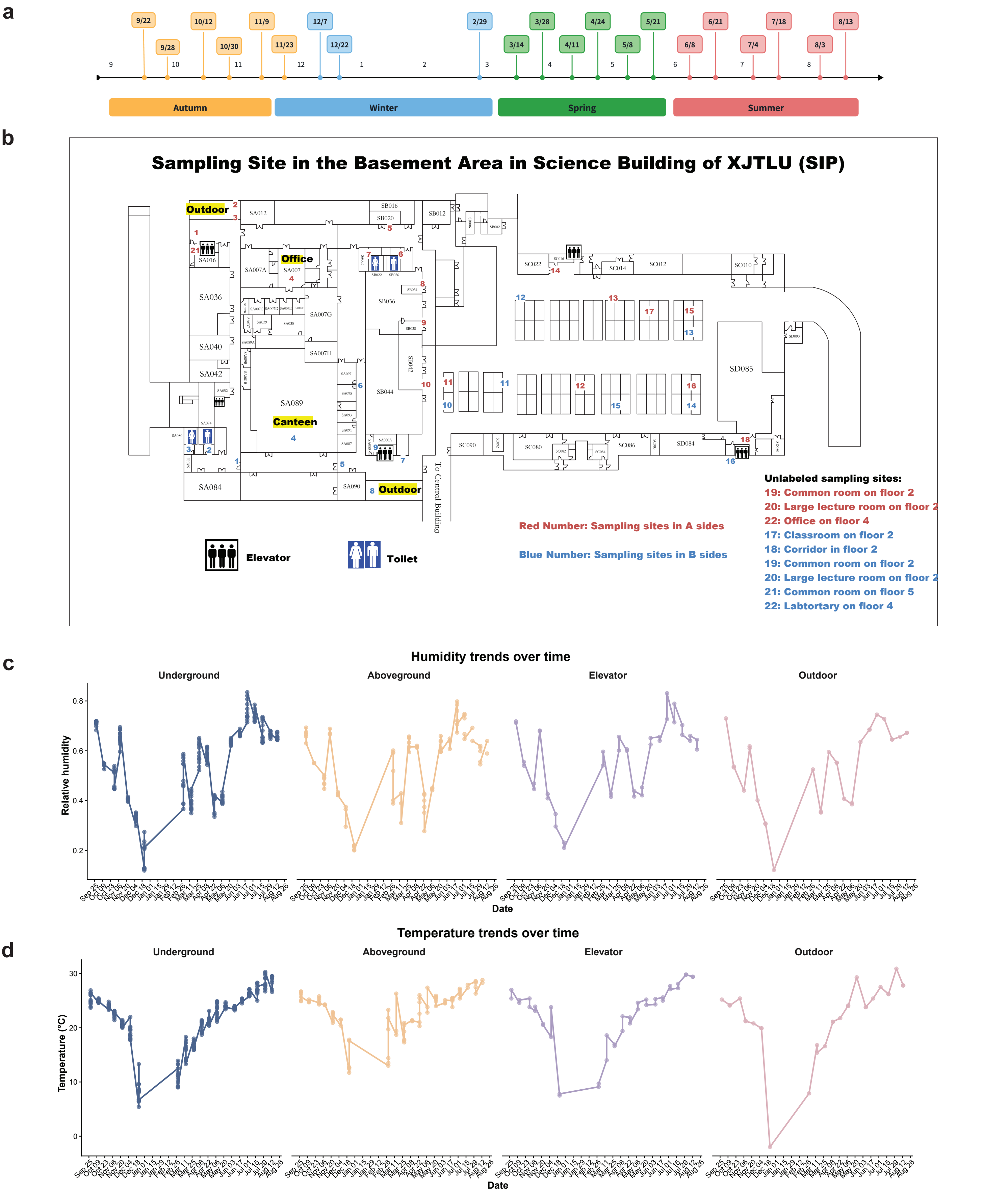

Figure S1. Overview of the spatiotemporal sampling strategy and environmental microclimates during the study period. (a) Chronological timeline of the sampling campaigns, illustrating the specific data collection points mapped across four continuous seasons. (b) Schematic floor plan detailing the spatial distribution of sampling sites within the representative underground environment. Distinct functional zones (e.g., elevators, offices, canteens) and specific sampling nodes are marked to capture the spatial configuration of the built environment. (c) Variation in mean relative humidity and temperature fluctuations were continuously monitored across the four defined spatial types and sampling dates.

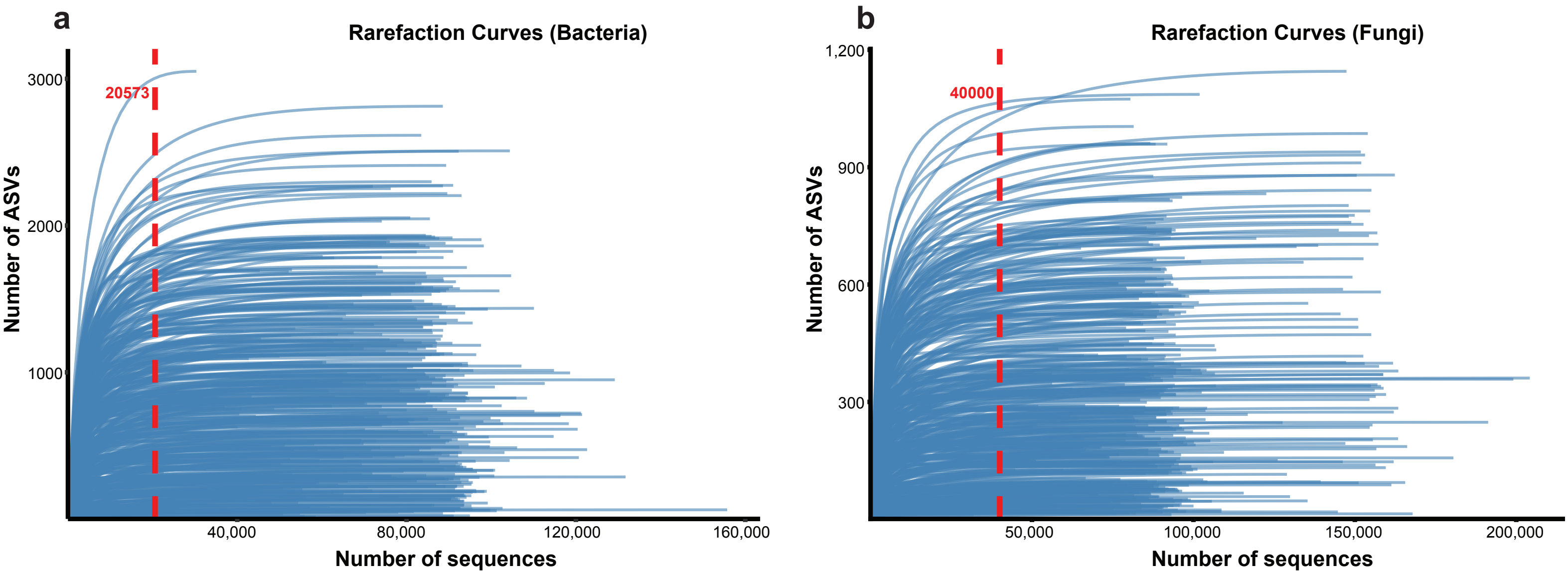

Figure S2. Rarefaction curves for microbial datasets. a) Bacterial datasets with a uniform rarefaction depth of 20,573 reads. b) Fungal datasets with uniform rarefaction depth of 40,000 reads. Curves per sample with a vertical dashed red line indicating uniform rarefaction.

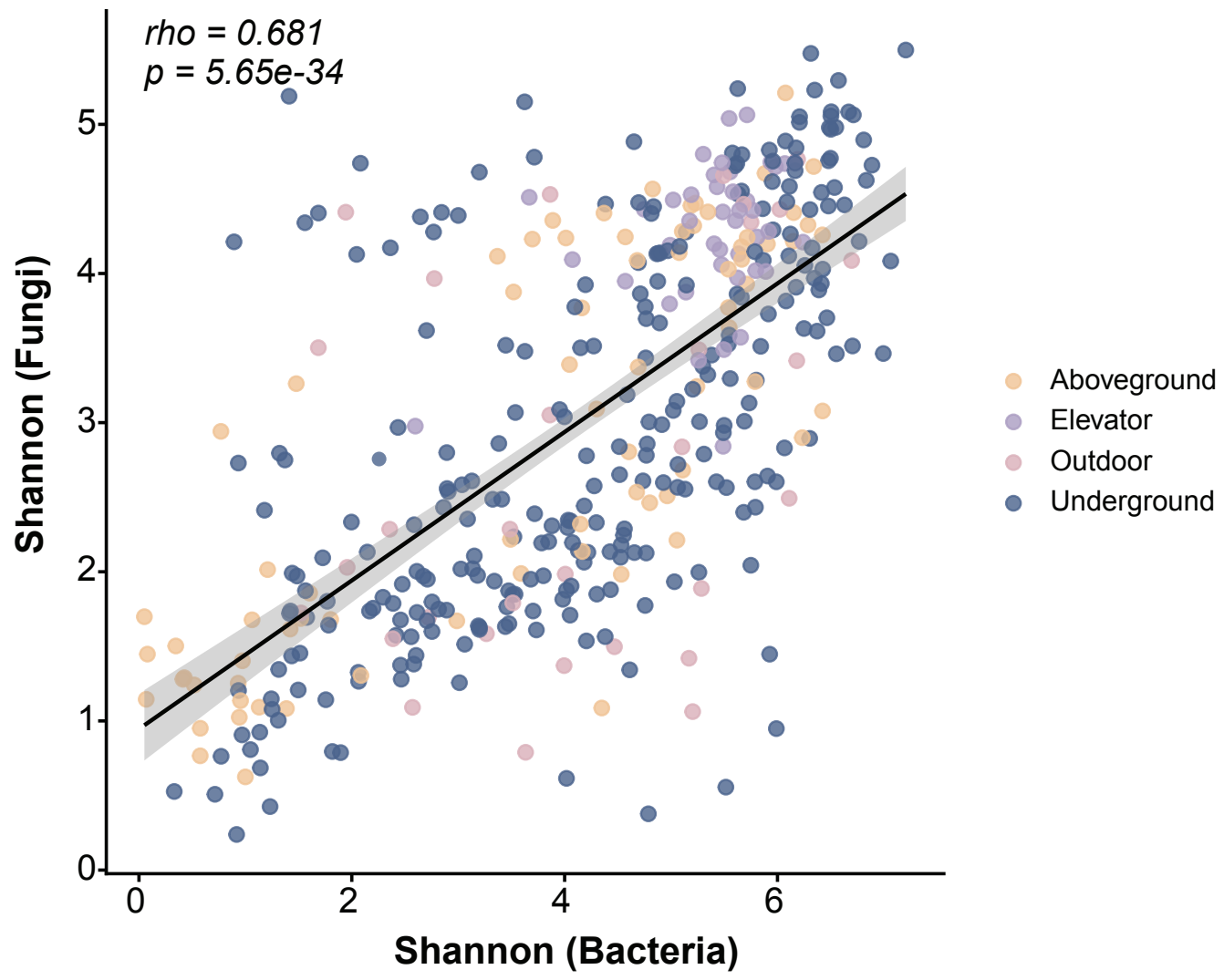

Figure S3. Spearman correlation between bacterial and fungal Shannon index.

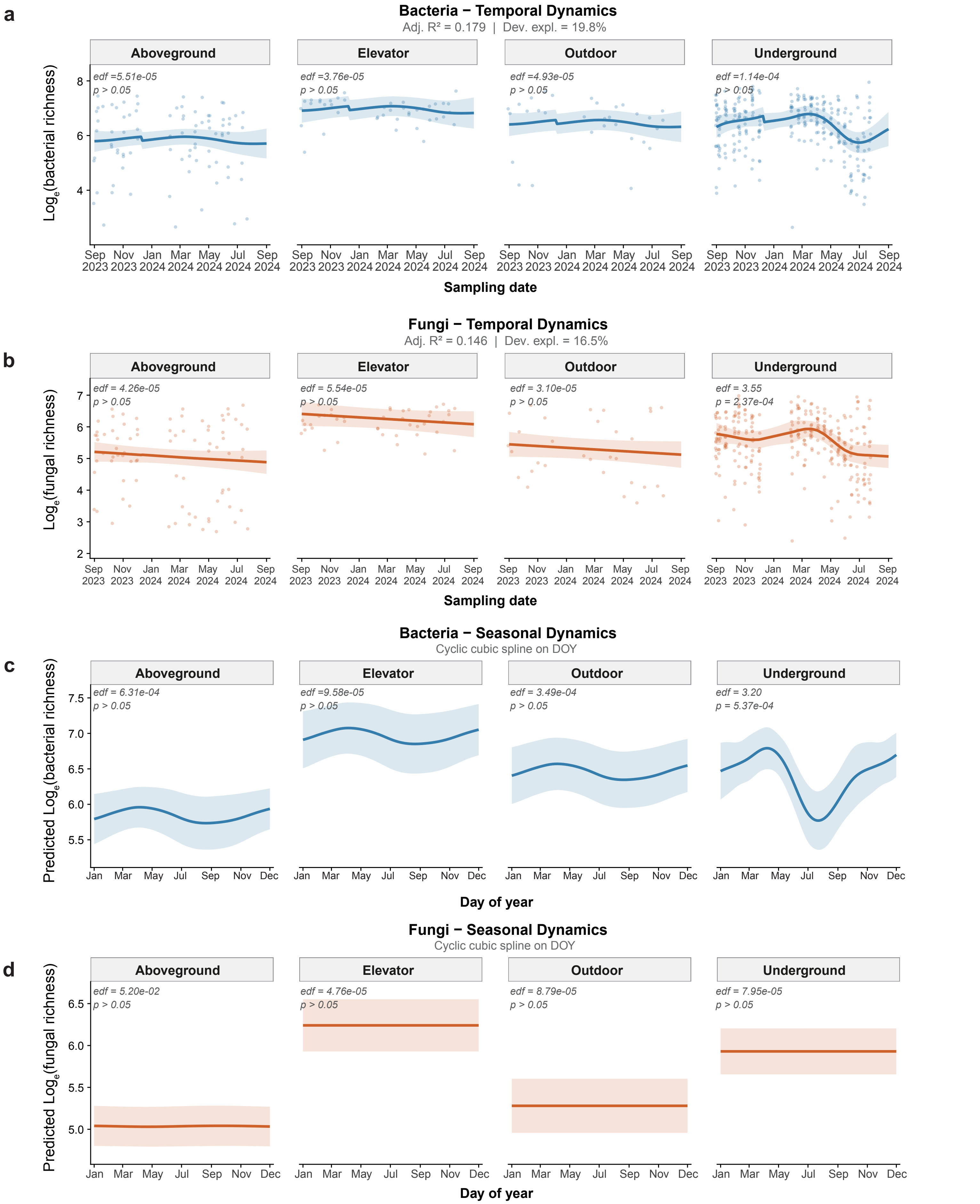

Figure S4. Seasonal dynamics of microbial richness inferred using generalized additive models (GAMs). a, b) Observed log-transformed richness of bacterial (a) and fungal (b) communities over time. Solid lines indicate GAM fits, with shaded areas representing 95% confidence intervals. c, d) Estimated seasonal trends derived from cyclic splines, showing annual periodicity while controlling for long-term trends.

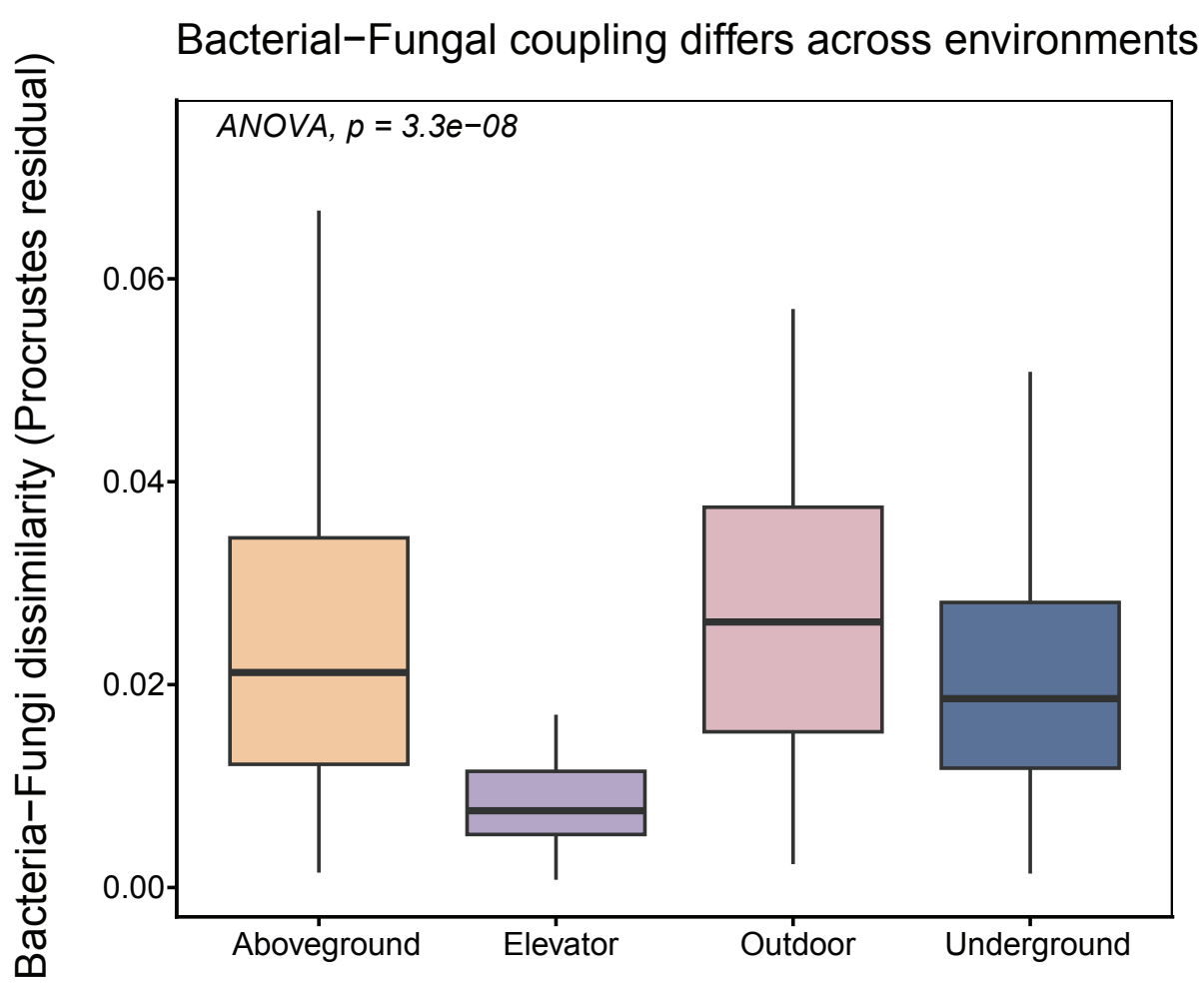

Figure S5. Boxplot of Procrustes residuals from the analysis of bacterial and fungal community dissimilarity.

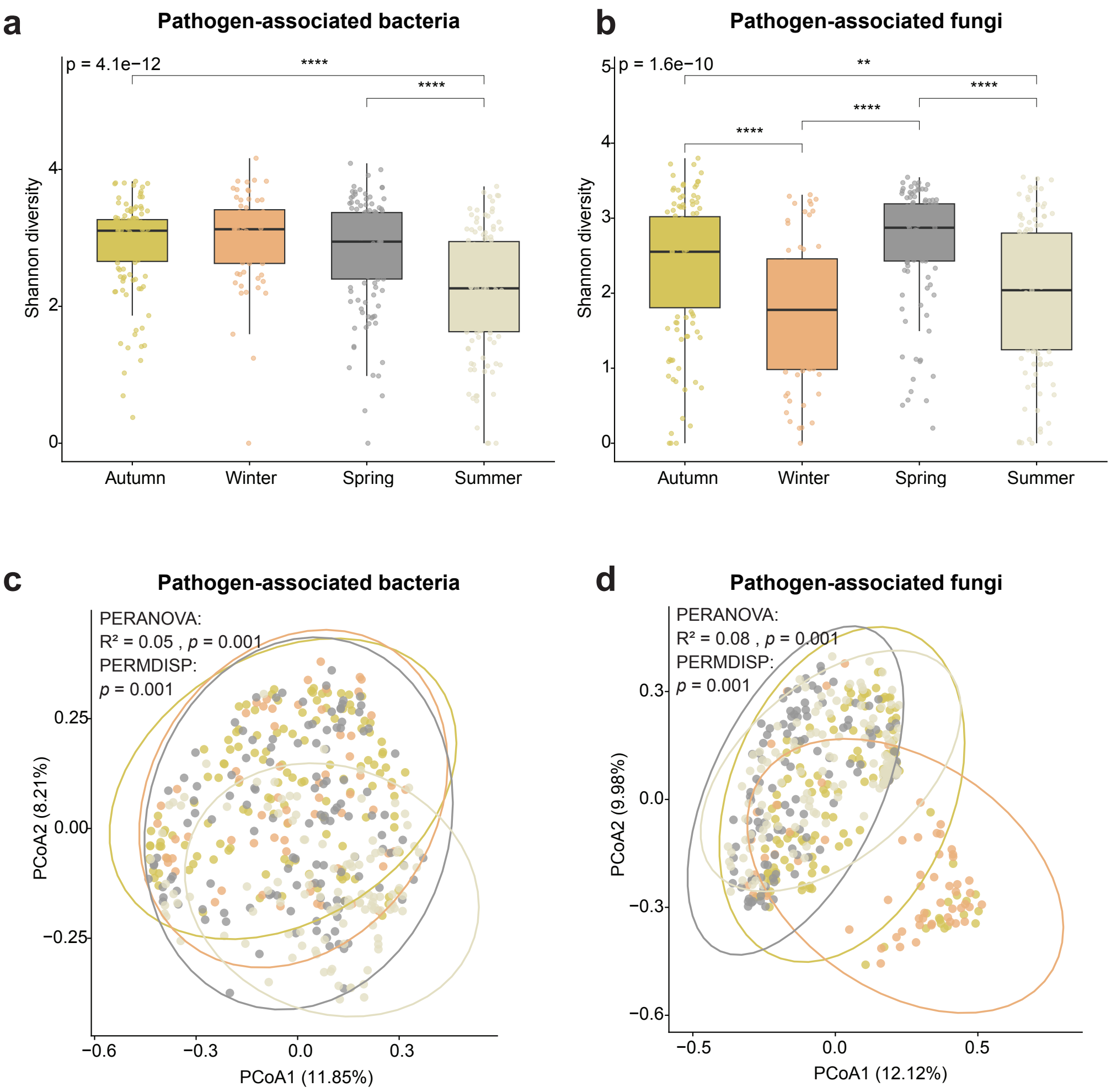

Figure S6. Diversity analyses for bacterial and fungal opportunistic pathogen-containing genera in different seasons. a, b) Shannon diversity of bacterial (a) and fungal (b) pathogen-associated communities across four seasons. c, d) Principal coordinates analysis (PCoA) of the pathogen-associated communities in four seasons, using Bray–Curtis dissimilarity for both domains.

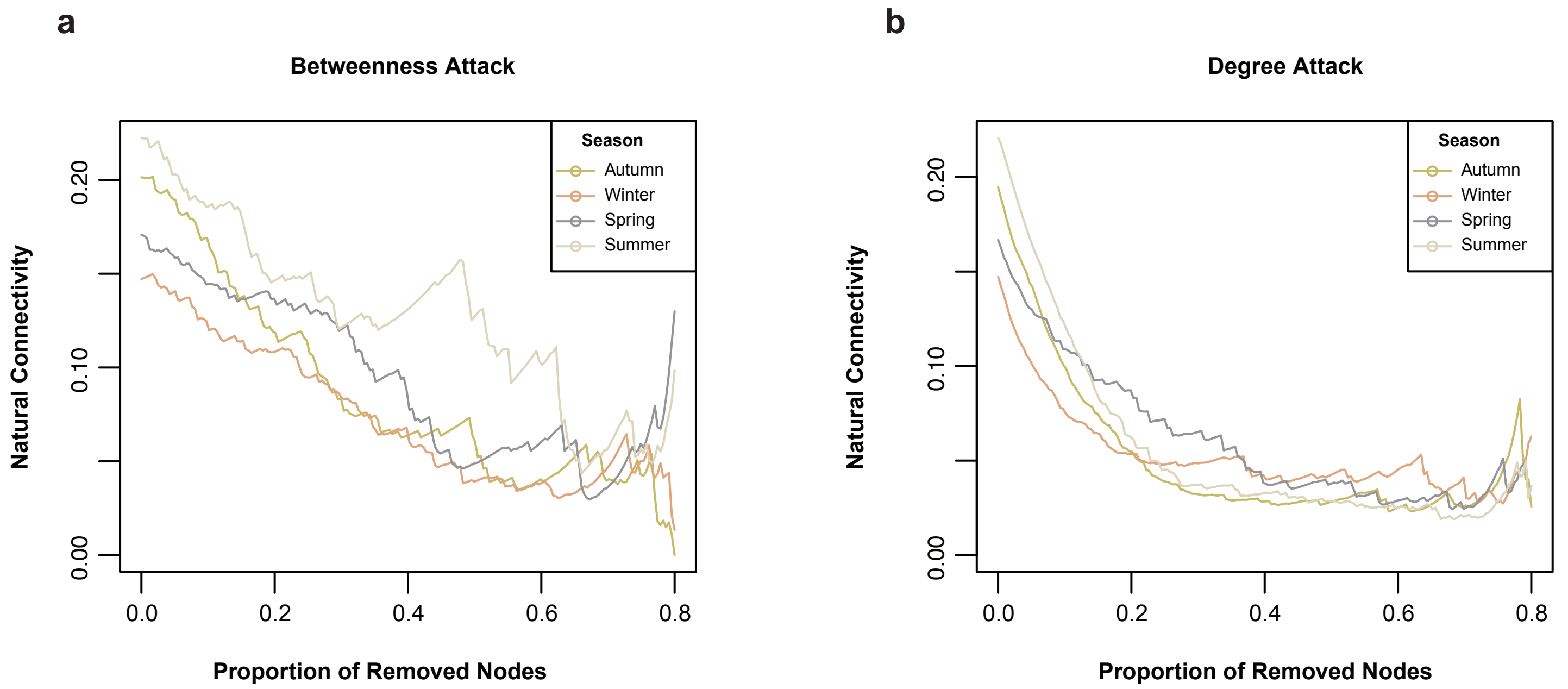

Figure S7. Seasonal variation in cross-domain network stability. a, b) Network robustness in each season was assessed by natural connectivity under targeted node removal. a) Betweenness-based attack: nodes were sequentially removed in descending order of betweenness centrality in the networks. b) Degree-based attack: nodes were removed in descending order of node degree in the networks. Lines represent the change in natural connectivity as a function of the fraction of nodes removed, with higher values indicating greater network stability.

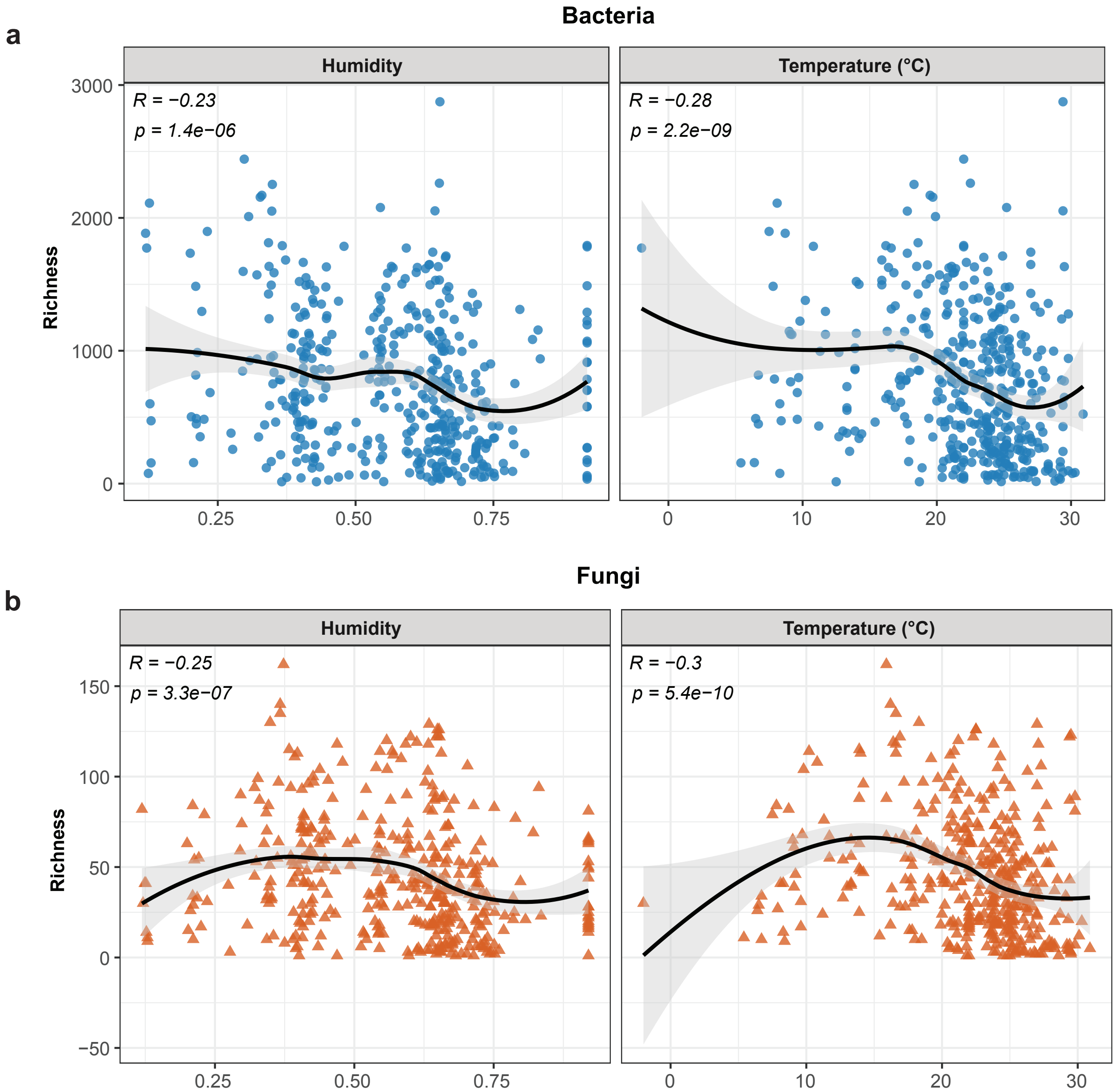

Figure S8. Associations between microbial richness and environmental variables. a, b) Spearman correlations between microbial richness and environmental variables, including temperature and relative humidity, for bacterial (a) and fungal (b) communities. Correlation coefficients (ρ) and significance levels are shown for each relationship.

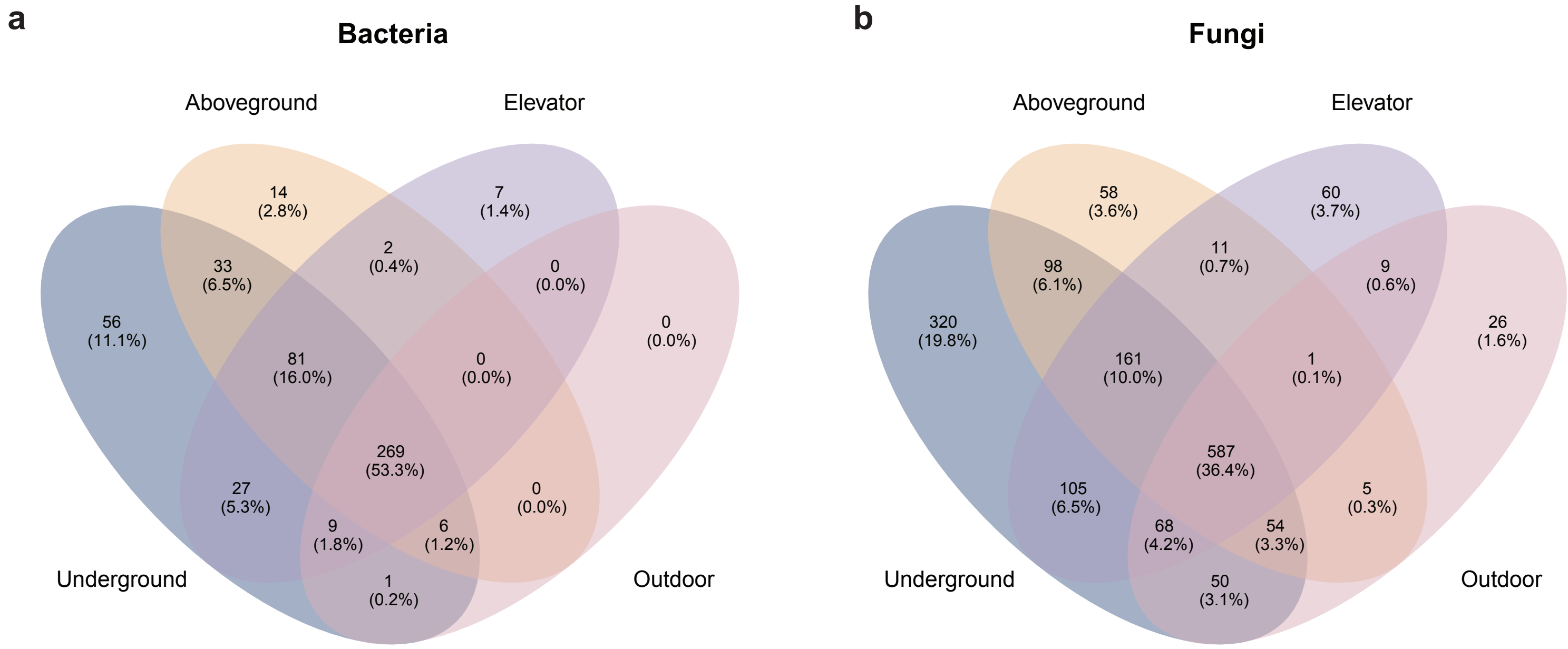

Figure S9. Shared and unique microbial genera across four spatial environments. a, b) Venn diagrams showing the overlap and uniqueness of bacterial (a) and fungal (b) genera among the four spatial groups. Numbers indicate the count of genera shared among or unique to each spatial environment.
